# A survey on the potential contribution of Reunion Island dye plant species diversity to the market demand for bioactive plant-based dyes and pigments

**DOI:** 10.1186/s13002-023-00580-w

**Published:** 2023-03-25

**Authors:** Mahery Andriamanantena, Shamsia Pithon, Manon Dijoux, Marine Hoareau, Christian Fontaine, Johnny Ferrard, Christophe Lavergne, Thomas Petit, Yanis Caro

**Affiliations:** 1 Laboratoire de Chimie et Biotechnologie des Produits Naturels (CHEMBIOPRO), Université de La Réunion, La Réunion, France; 2 Conservatoire Botanique des Mascarins, Saint Leu, La Réunion, France; 3 Département Hygiène Sécurité Environnement (HSE), IUT de La Réunion, Université de La Réunion, La Réunion, France

**Keywords:** Ethnobotany, Reunion Island, Dye plants, Pigments, Natural colorants

## Abstract

**Background:**

Proven toxicity and environmental burdens caused by artificial dyes have motivated dyeing industries to turn to natural alternatives. Plant-based dyestuffs are an interesting group of alternative crops. Reunion Island located in the Indian Ocean is the only European region in the southern hemisphere. It has a great number of assets to find new molecules in the abundant plant biodiversity. However, the dye-producing plants diversity in this island had not been documented to date.

**Methodology:**

The assessment of the Reunion Island’s plant biodiversity through the “PLANTIN” project allowed us to establish here the first ethnobotanical inventory of plants growing on Reunion Island which may have promising properties as a new alternative source of dyes or colorants for the industries. First, an ethnobotanical survey focused on the uses of plants traditionally used in dyeing was conducted on local stakeholders. Then, the importance of different criteria (*e.g.*, endemicity, accessibility and cultivability, plant organs used for the extraction, industrial interests of the species, etc.) has been considered to establish a classification method of the species, to finally select the most interesting plants which have been further harvested and investigated for their coloring property and dyeing application on natural fibers.

**Results:**

The results showed that local people have accumulated traditional knowledge of dyeing plants, but that this approach had been discontinued in Reunion. The uses of **194** plant species potentially rich in dyes or pigments, belonging to **72** different families, with diverse botanical status (endemic, native, introduced or alien-invasive species) have been recorded. Then, 43 species were harvested and their coloring property were investigated. It demonstrated that dyes extracted from promising species, *e.g*., *Terminalia bentzoe, Weinmannia tinctoria, Thespesia populnea, Erythroxylum laurifolium, Morinda citrifolia, Leea guinensis, Ochrosia borbonica, Danais fragrans, Terminalia cattapa, Casuarina equisetifolia,* and *Coccoloba uvifera*, amongst others, could be used as new textile dyes. Their efficacy in the wool and cotton dyeing has been successfully demonstrated here.

**Conclusion:**

These plant-based dyestuffs showed promising coloring properties with different shades that could meet industrial application requirement. It's an area that could promote local cultural inheritance, create opportunity for business and farmers, and that can make a significant contribution to preserving endangered native species by supporting reforestation schemes. Additional researches are in progress to evaluate the safety of these plant-based colored extracts, their chemical composition and biological activities.

**Supplementary Information:**

The online version contains supplementary material available at 10.1186/s13002-023-00580-w.

## Introduction

The hazardous effects of artificial dyes on human health like carcinogenic or mutagenic activity [1], and the contamination of the environment caused by wastewater discharged by dye industry [2] led the industries to search new sustainable and less harmful dye sources [3]. Natural dyes are more environment-friendly than artificial dyes and may have biological benefits [4]. Therefore, the natural dye market segment has emerged and has become increasingly important in global financial markets and linked with other important global issues. The global dyes and pigments market size was estimated from US dollars (USD) 29 to 36 billion in 2021, whereas the global market of naturally derived dyes and pigments (mineral and organic pigments) was valued at USD 5 billion, *i.e.*, from 14 to 17% of the value of the global market [5]. The European market of natural dyes and pigments is valued at USD 1 billion in 2021, and the plant-based dye and pigments represented 70% of this European market in terms of financial income, *i.e.*, USD 559 million. France, the third largest European producer of naturally derived dyes and pigments, after Germany and Italy, has a production of 49.4 million euros (9% of the entire production of natural dyes and pigments in this European market) [5]. However, despite the high demand from consumers, the natural dye industry faces some technical and sourcing issues. The application of natural dyes remains limited because of their somewhat restricted color range, low levels of color-fastness compared with artificial dyes, low levels of purity, low yield, instability and sensitivity to many environmental conditions, the raw material availability and the high cost of their production [6]. To overcome these limits, researches for more sustainable products should involve the development of environmentally friendly production processes and the exploitation of new alternative sources of colorants, such as microbial pigments which become interesting by their sustainable production, biodegradable and coloring properties [7, 8].

Some plants are excellent raw materials for producing natural colorants as they have a great amount of water-soluble dyes or plant pigments, like dye plants such as indigo and madder [9], while others just don't seem to have enough pigments. Dye-producing plants possess a wide spectrum of utilization and they are an interesting group of alternative crops, providing that they will be cultivated and exploited in a sustainable way [6]. Some natural colorants, such as carotenoids, flavonoids, hydroxyanthraquinones, etc., extracted from plants are promising dyestuffs and have regained popularity in a wide range of industries. They are widely used as coloring bioactive substances in several manufactured products, including textiles, cosmetics, food, pharmaceutical, plastics, paint, ink, paper and electronics *(e.g*., for dye-sensitized solar cells) industries [4, 9, 10] due to their optical properties and additional biological (*e.g.*, antioxidant, antibacterial, antiviral, and antifungal) activities [11–13]. Thus, the coloring property as well as the added nutritional or therapeutic potentialities of some plant-based dyes and pigments make the biomaterials an important agricultural operation that have the potential to face the challenges of substitution of artificial colorants with the colored biocomponents extracted from different plant parts (seeds, fruit, flowers, stems, barks or adventive roots).

Dye plants can be found in a wide range of vegetation types, growing areas and climates, like in temperate forests and tropical forests. The sustainable exploitation of selected dye plants would make it possible to palliate certain diseases and environmental pollutions induced by the use of artificial colorants, to improve the agricultural income of the local populations practicing its culture, to create opportunity for business and farmers, and to offer to the selected dye-plant species a visibility for agricultural valorization of their extracted colored biocomponents [14].

From that perspective, Reunion Island located in the southwest of Indian Ocean is a good candidate as it counts among the world’s top biodiversity hotspots with an endemic rate approximately of 40% [15]. Its plant biodiversity results from the colonization of species from Madagascar, Africa, Asia, India and Australia. For instance, many indigenous species from Reunion Island have been demonstrated to have medicinal properties and around 20 species have recently been incorporated in the French pharmacopeia [16]. Nevertheless, to the best of our knowledge, the dye-producing plant species diversity in Reunion Island had not been documented to date. No specific data are available on plants potentially rich in dyes or pigments in this island.

Hence, this original research study aims, for the first time, to inventory all the dye-producing plants or plant species potentially rich in dyes or pigments from the endemic, native, introduced and alien-invasive plants growing in Reunion Island. Firstly, an ethnobotanical survey focused on the uses of plants traditionally used for dyeing in Reunion Island was conducted. Then, we developed an assessment scoring system that used different criteria to evaluate and assign each plant a “dye score,” to finally select the most promising species which have been further harvested from eight growing areas in Reunion Island and investigated in detail for their coloring property and application in textile dyeing.

## Materials and methods

### Study area

Reunion Island (55° 3′ E and 21° 5′ S), with Mauritius and Rodrigues, is one of three islands that composes the Mascarenes located in the southwest of Indian Ocean. It is the only European region in the southern hemisphere. Reunion Island is a French overseas department and divided into 116 municipalities. The population is mainly composed of people originated from France, Africa, Madagascar, India and China. This is a relatively small tropical island, approximately 2500 km^2^. Economic activities and about 80% of the population are located in the coastal lowlands due to the rugged topography of the island which was created two million years ago by the emergence of a submarine volcano. This particular formation has been considered as the main factor that give to this island the high endemism of growing plant and animal species [15]. Almost 40% of the territory belongs to the National Park, which was established to preserve and conserve the terrestrial biodiversity.

### Exploration of the Reunion island dye plant species biodiversity

The ethnobotanical survey of the dye plant species biodiversity of Reunion Island was conducted by botanists of the Conservatoire Botanique National des Mascarins (CBNM) in Reunion island. A literature review was conducted by consulting the available documents on the history and plant diversity of Reunion Island at the University of Reunion and the CBNM libraries. The works of Boullet et al. (1886, 2020) in “Index des Tracheopytes” [17], Jacob de Cordemoy (1895), the “Flore des Mascareignes” written by Bosser et al. (1935, 1998, 1999, 2011) [18–20] or Lavergne (1990, 1994, 1996) [21, 22], amongst others, allowed us to inventory most of the Reunion Island dye-producing plant species or plant species identified as potentially rich in dyes or pigments. Several online documentation and database focused on ethnobotany, chemistry, biodiversity, dyes and pigments or medicinal plants have been as well consulted. Furthermore, all the information obtained from the reviewed literature has been combined and supplemented by information obtained from the ethnobotanical survey of Reunion Island’s dye plants conducted by a team leaded by Christophe Lavergne from CBNM. This survey from January 2021 to November 2022 relied on the local peoples and stakeholders who have a good knowledge of the history and culture of Reunion Island including local dyers, farmers, small cosmetics manufacturers, botanists, etc. Ethnobotanical data were recorded via face-to-face interviews in the local language (Créole) or French, depending on the interviewee. The questionnaire concerned the knowledge of the traditional uses of some plant species as coloring materials, and the survey enabled us to inventory additional plant species traditionally uses in Reunion Island for natural dyeing. Ten questions, listed below, have been asked to each stakeholder: “1/Do you know any dye plants, i.e., plants that are sources of pigments or dyes? 2/Are you familiar with natural dyeing? Yes/No 3/If yes, which ones do you know? 4/According to you, which ones have been traditionally used in Reunion Island? 5/According to you, what are the parts of the plant used for each species? 6/In your opinion, what are the collection methods and have these plants been cultivated? 7/According to you, what are the extraction methods and preparations that were or are currently used for dyeing? 8/What are the different applications where dyeing from plants has been used: tanning, dyeing, medicinal use, food, crafts? 9/Was there a trade of the extracted substances and if so, according to which economic model? 10/Do you know any references or literature sources or people describing dye plants?.”

### Scoring and evaluation of the most promising plant species for coloring applications

Following the assessment and survey of dye plant biodiversity, we developed an assessment scoring system that used different criteria to evaluate and assign each plant a “dye score” to determine the most promising plant species from Reunion Island for coloring applications. Using a scientific approach to the dye score, we established eight criteria and four indexes, which can be described as follows. The first six criteria (criteria 1 to 6) are based on empirical knowledge collected from the literature review and ethnobotanical survey of the considered plant species. The other two criteria (criteria 7 & 8) are based on laboratory experiments using a standardized, eco-friendly extraction process of dyes from the raw materials as previously described by the authors [14]. Each criterion is ranked on a scale from 1 to 10, where 1 is the lowest score meaning that the plant species has no or very weak interest related to this criterion and 10 is the highest, indicating that the species exhibits great potential related to this criterion. We have classified each criterion according to the “dye score” ranking definition developed in Table 1.

**Table 1:** Notation of the 8 criteria for scoring the "dye score" of the plant species from La Réunion Island biodiversity

Criterium	Description	Notation of the criterium on a scale of 1 to 10			
		1	3	7	10
C1—Indigenous status of species on La Réunion Island	Valued endemic plants because of their potential as a locally available resource that can lead to regional economic development in addition to the extension and conservation of biodiversity	Introduced (alien)	Native or cryptogenic	Regional endemic (Mascarenes)	Strict endemic of Reunion Island
C2—Scientific knowledge about species	Valued the identification of species not previously described in the scientific literature, nor enhanced in the industrial field	Species perfectly described in literature and pigments identified (known structure)	Species relatively well described in the literature	Some references in literature but pigments not known	No article in the studied species
C3—Accessibility and availability of species on La Réunion Island	Valued locally available plants that have a high economic potential for the territory. It is essential to prioritize readily accessible species that are not protected in Reunion	Protected and rare species (with difficult access)	Species accessible with permission: private land, departmental—domain, national park, but with enough plants available for collection	Abundant species in the wild with relatively easy access (no resupply problems)	Abundant species in cultivation and easy access: both in cultivation (CBNM arboretum) and in the wild (EEE), no resupply problem
C4—Cultivability of plant species	Valued cultivability to elevate species already cultivated on the island and plants whose reproduction and cultivation methods are known	Not cultivable	Cultivable but very slow growing (e.g., 10-year-old tree)	Potentially cultivable from its reproduction mode	Cultivable and currently cultivated in La Réunion
C5 – Plant organs used for pigment extraction	Valued sustainability by prioritizing use of the most renewable parts of the plant. Leaves can be harvested throughout the year, bark can be collected if it does not affect the future development of the plant. Flowers and fruits are seasonal but if the yield is high, these parts have potential. The collection of the woody part of a plant can kill it and are therefore not sustainable	Wood, all plant	Adventitious roots	Stems, barks, fruits, flowers	Leaves
C6—Industrial interests and other known applications of plant species	Valued other medicinal, biogas, nutritional or other uses beyond the coloring properties of the plant—greater economic interest	No other known uses apart from traditional dyeing	1–2 other known uses	3–4 other known uses	Multiples known uses > 5
C7—Color and stability of dyes and pigments extracted from plant species	Valued rare/special colors (blue, magenta, pink, cyan, mauve, black…). Yellow, orange and red are sought in the dyeing industries to replace synthetic dyes, but only if they have an intense and stable color. Brown and beige colors are less demand	Colorless or very light-colored extract	Unattractive (beige, brown, etc.) and/or unstable color	Desired color (red, orange, yellow…) and relatively stable	Rare/special and relatively stable color (blue, magenta, pink, cyan, mauve, black…)
C8—Yield and difficulty of color extraction	Valued plants containing water-soluble pigments and dyes that are easily extractable using water and ethanol (eco-compatible solvents) with adequate yield for industrial applications, and whose color can be realized without mordanting or fermentation	Mordanting or fermentation useful for color expression	Pigments extractable only with non-eco-compatible solvents (e.g., hexane)	Pigments extractable with EtOH aqueous in average yield	Pigments extractable with EtOH aqueous in good yield

*Criterion 1 (C1)* represents the “endemicity level” of the considered plant species on Reunion Island. On a scale of 1 to 10, the lowest score 1 was given to exotic and introduced species; 3 to the native and cryptogenic plants; 7 to the Mascarenes regional endemic species in the Indian Ocean; and 10 was given only to the Reunion Island endemic species.

*Criterion 2 (C2)* represents the scientific knowledge currently available on the considered plant species. As this assessment and survey of the dye plant species biodiversity of Reunion Island is intended to be innovative, the highest score 10 was given to the plant species which are still unknown to the scientific community (*i.e.*, including those that are undescribed or are described but otherwise data deficient in literature). The lowest score 1 was given to perfectly described species with botanical and chemical data (known pigments, etc.). The intermediate scores 7 and 3 were given to relatively described species, with or without known information on coloring properties of the species.

*Criterion 3 (C3)* evaluates the accessibility and availability of the considered plant species in Reunion Island. Taking a conservation bio-diversity-centered approach, critically endangered, rare and/or protected plants that are difficult to access were given the lowest score 1. A score of 3 was given to the unprotected species that are accessible in protected areas with authorization; 7 to species that are abundant in the wild; and 10 to species currently cultivated in Reunion Island.

*Criterion 4 (C4)* represents the cultivability. A score of 1 was given to plants that are very difficult to grow; 3 to potentially cultivable but slow-growing plants; 7 to known cultivable plant; and 10 to the currently cultivated plants.

*Criterion 5 (C5)* ranked the botanical parts used for pigment extraction on a scale of 1 to 10, privileging the most renewable parts of plants. Thus, a score of 1 was given to the whole plant or the trunk, the harvesting of which would most likely kill the plant; 3 to the adventitious roots; 7 to the barks, fruits and flowers; and 10 when the leaves are used for coloring applications.

*Criterion 6 (C6)* ranked the industrial interests and known applications of the plant species, where 1 was given to the plants without any other known interests or uses (apart from their tinctorial interest); 3 to plants with one or two other known uses; 7 to the plants with three of four other uses; and 10 to the plants with more than five other known interests or uses.

*Criterion 7 (C7)* evaluated the color and stability of the dyes and pigments extracted from the plant species. Some colors are more interesting than others in terms of industrial applications, so they were assigned a higher score. A score of 1 was given to colorless extract obtained from the standardized dye extraction described below; 3 to the common brown, black, or beige hues or for a non-stable color; 7 to red, orange and yellow hues; and 10 for stable and unusual colors like blue, green and pink.

*Criterion 8 (C8)* rated the yield and difficulty of extraction. The highest score of 10 was given to dyes and pigments that were extracted easily and efficiently using the eco-extraction method with water and ethanol as solvents; 7 to plants containing pigments that are easy to extract with a mixture of water and ethanol but with a low yield (< extraction yield mean value for each collected plants part, w/w); 3 to plants containing pigments that are insoluble in a mixture of water and alcohol, or pigments that are difficult to extract using this sustainable process and therefore require non-eco-compatible organic solvents; and 1 to dye plants that need a transformation to express the coloration (*i.e.*, by mordanting or fermentation).

Finally, four indexes were further calculated based on these eight criteria: (1) The “*Endemicity index*” (${\text{Iend}}$) was calculated by multiplying criterium C1 × C2; (2) The “*Cultivability index*” (${\text{Icult}}.$) was calculated by multiplying criteria C3 × C4; (3) The “*Industrial extrapolation index*” (${\text{Iind}}.$) was calculated by multiplying criteria C5 × C6; and (4) The “*Coloring strength index*” (${\text{Icol}}.$) was calculated by multiplying criteria C7 × C8. We have classified each index according to the “dye score” ranking definition developed in this study. Given that the hues, the yields and the difficulty of the color extraction were the most important criteria for coloring applications in industries, the “*Coloring strength index*” was counted two times more than the other indexes for each plant species final “dye score.” This “dye score” is calculated using the following formula:

1 $${\text{Dye score }} = \frac{{\left( {{\text{Iend}}.{ } + {\text{ Icult}}.{ } + {\text{ Iind}}.} \right){ } + { }\left( {{\text{Icol}}.{\text{ x }}2} \right)}}{5}$$

where *Endemicity index (*${\text{Iend}}$.) = C1*C2; *Cultivability index (*${\text{Icult}}.)$ = C3*C4; *Industrial extrapolation index (*${\text{Iind}}.)$ = C5*C6; *Coloring strength index (*${\text{Icol}}.)$ = C7*C8; and C1 is the endemic status, C2 is the scientific knowledge, C3 is the accessibility and availability, C4 is the cultivability, C5 is the botanical parts used for pigment extraction, C6 is the Industrial interests and other known applications of the species, C7 is the color and stability of dyes and pigments extracted, and C8 represents the yield and the difficulty of the color extraction.

### Plant material collections

**Fig. 1:**
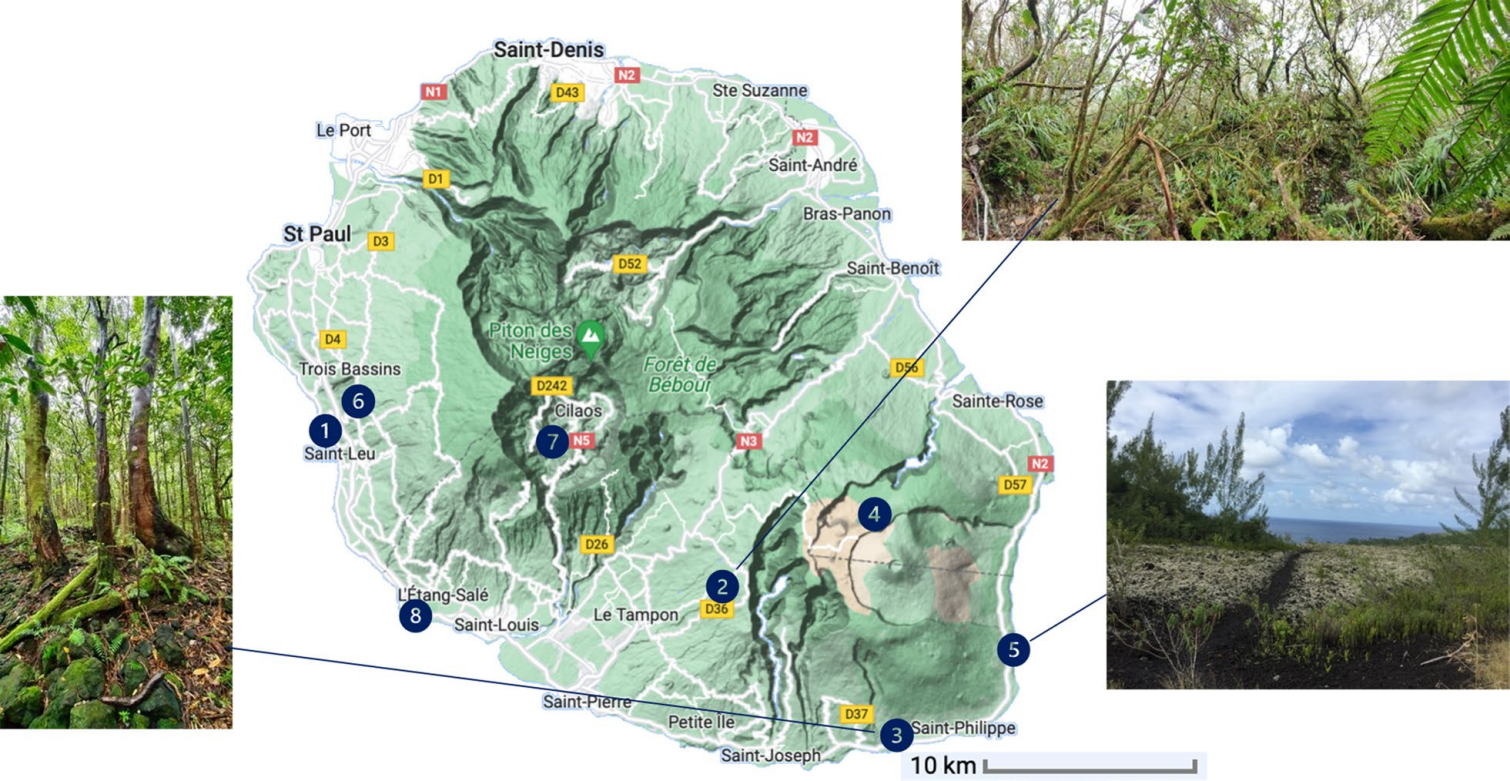
Map of Reunion island, with some plant collection points (1: Ravine des Colimaçons, 2: Notre Dame de la paix forest, 3: Mare Longue forest, 4: Piton Partage, 5: Tremblet, 6: National Botanical Conservatory of the Mascarene, 7: Cilaos, 8: Étang-Salé) (*Source: Google map*)

Around 60 interesting dye plant species selected from our assessment scoring system have been further harvested in different areas of Reunion Island as shown in Fig. 1. The collection of plant species growing in protected areas or territories of the national park has been validated by the competent authorities under the *authorization* N° DIR/SEP/2021/144 and DIR-I-2021-335. Identification and conservation of each species harvested have been made by botanists of the CBNM. Voucher specimens have been deposited in the herbarium of the CBNM. The CBNM collection form was used for the traceability of each collection. Depending on the species, adventitious roots, barks, stems, leaves, flowers, fruits or lianas were collected. The harvesting was done according to good practices in order to reduce adverse effect on the plants. For example, in the case of bark and roots, a part was scraped off and then pine tar was sprayed on the cut to avoid external contamination. The quantity of fresh material to be collected varies according to the part of the plant concerned. Thus, the ideal proportions to be collected to ensure a sufficient quantity of fresh material for pigment extraction were estimated at 500 g for barks and roots, 1 000 g for leaves and flowers, and 2 000 g for fruits. Plant organs were collected from three different trees or shrubs from the same geographical and geological area, and they were then mixed to constitute one representative collection batch. The collection was repeated three times.

Each collection batch undergoes various pre-treatments before pigment extraction, including cutting, drying and grinding. All samples were firstly cut into small pieces. Two different drying methods have been used: freeze-drying (Labconco freezone2.5) used in the case of fruits (due to high moisture content and nutrients that may favor the development of molds during slow drying), and for drying leaves, bark or roots with low moisture content, the drying was carried out at 45 °C using a climatic chamber (Memmert HPP410) with a relative humidity controller set at 10% in order to achieve a slow drying process to avoid damage on the plant like browning and chemical reactions, due to a high temperature. Duration of the drying process varies according to the initial moisture content, generally ranging from a few days to a week. Then, the sample was coarsely ground with a mill (Retsch SM100) and finely ground with a laboratory mixer (Kinematica Microtron MB550) to finally obtain a powder that will be kept away from light and humidity at 4 °C before the extraction stage.

### Pigment extractions and evaluation of coloring properties of the plant-based dyestuffs

Pigment extraction have been performed using the methods described previously by the authors [14]. Briefly, a pressurized liquid extraction (PLE) under nitrogen was applied with a mixture of ethanol and water (70/30, v/v) by using an accelerated solvent extractor (ASE™ 350, Accelerated Solvent Extractor, Dionex). About 10 g of dry plant materials are mixed with diatomaceous earth to fill a 34 ml ASE cell. Conditions were as follows: temperature, 45 °C; pressure, 110 bars; three cycles with static extraction time of 3 min; purge time 2 min; and flush volume 100%. The procedure was performed three times under repeatability conditions. Then, the crude extracts were concentrated on a centrifugal evaporator (Genevac EZ2 +) at 45 °C under vacuum, resolubilized on a small volume of the same extraction solvent to be finally lyophilized (Labconco freezone2.5) during 48 h in order to generate crude dry color extract in powder (*i.e.*, the plant-based dyestuff) for pigment extraction yield calculation. All extracts were kept at 4 °C until further use.

To check the coloring property of the dry color extracts, the sample was dissolved on the same solvent used for the extraction at different concentrations, *i.e.*, from 1.0 to 32 mg/mL of dry powder extract in solution, to show the evolution of the shade of the sample by their color value measured in the international CIELab color system in terms of L* (lightness), a* (greenness versus redness), and b* (blueness versus yellowness). The color coordinates were analyzed using a spectrophotometer (Konica Minolta CM-3600A) with 10 mm pathlength quartz cuvette, combined to the color data software SpectraMagic NX (Konica Minolta) for data analysis.

Furthermore, optical density of the sample has been measured with a spectral scanning from 200 to 800 nm by an UV–visible spectrophotometer (Thermo Scientific Genesys 10S). The same solvent used for the extraction and dilution was used as a blank. To check the solubility in water and the stability of the colored extracts toward pH and temperature variations, materials were dissolved at 4 mg/ml in Britton-Robinson water buffer solutions of pH 4, pH 7 and pH 10, and then exposed to different temperatures from 25 to 95 °C using a water-bath. The exposition time was from 4 to 24 h.

Then, the dyeing property of the plant extract has been checked for bath-dyeing wool and cotton fibers with different mordants: 1-potassium alum + potassium bitartrate mordant; 2-aluminum acetate mordant; 3- aluminum acetate mordant + tannins (gallnut); 4- aluminum acetate + ferrous sulphate mordant; 5- aluminum acetate + tannins (gallnut) + ferrous sulphate mordant; 6-aluminum acetate mordant + ash lye + sodium carbonate.

### Statistical analysis

The statistical analysis for each experiment was performed using analysis of variance (ANOVA) and expressed as mean ± standard deviation of three replicates. The procedure was followed by Duncan test to determine the statistical significance (*p* < 0.05).

## Results and discussion

### Ethnobotanical survey of plants traditionally used in natural dyeing on Reunion Island

In mid-1600 s, the Reunion Island was first settled by French colonist. They bring slaves from Madagascar, Africa India, and Asia to work first in cane and coffee plantation. Slaves brought with them some medicinal and other plants traditionally used in their country. Progressively, many new plant species were introduced by people from all walks of life. People bring some traditional knowledge and know-how notably in the field of dyeing textile from Madagascar [23, 24], and some dye-producing plants were probably introduced at that time. The evolution of indigenous species, far from their region of origin, led to the emergence of several endemic species [25]. In this study, the assessment of the Reunion Island’s plant biodiversity through the “PLANTIN” project (co-funded by European Regional Development Fund) allowed us to establish the first ethnobotanical inventory of plants growing on Reunion Island which may have promising properties as a new alternative source of dyes or pigments for the industries.

Local people from Reunion Island has accumulated traditional knowledge of dyeing plants and their uses over the years. This approach had been discontinued in Reunion, in comparison with Madagascar, where a rich ancestral knowledge of dye plants has been described and transmitted from generation to generation [6, 14, 24]. The oldest trace of the uses of dye plants in Reunion Island seems to come from *Indigofera* plantations in the north of the island, which was abandoned a long time ago. The uses of *Curcuma longa* locally called “saffran péï” have been cited several times during the survey. Considering that this plant is very present in the Indian culture, it is not surprising to see the extent of this plant in different uses in Reunion Island including natural dyeing as his history is attached in great part to the Indian culture. The third most famous dye plants in the island is the famous endemic species *Weinmannia tinctoria* Sm., locally called “Tan rouge” (red “tan”) [26]. While bark of this last specie was cited several times in the literature review and during the ethnobotanical surveys as used traditionally for tanning application in Reunion, no scientific data are really available on its utilization for tanning applications over the world. Furthermore, there is evidence that this natural dyeing with plant-based textile dyes has not been abandoned uniformly in Reunion Island, particularly in recent years. Craftsmen and small companies working in textiles have tried to reintroduce dye plants from some alien-invasive species in Reunion Island into their dyeing process (*i.e.*, for the formulation of traditional dye baths in hot water). For instance, “WHOLE,” “Kouleur local,” and “Nuance de Couleurs” are examples of companies that are recently working on natural dyeing from some Reunion Island’s dyestuffs of plant origin. As the reuse of alternative sources of colorants including dye plants has grown considerably these last decades, this new trend can contribute to the conservation of some species as the example of *Weinmannia tincoria*. Actually, *W. tinctoria* was famous for its dyeing properties and known by most of the local population. Due to its non-use, it has been replaced by other species and has been considered as an endangered species lastly by the International Union for Conservation of Nature (IUCN). This a great example of the loss of some traditional knowledges in Reunion Island and the importance of its conservation as it can also preserve some species to not disappear. If it will be used as dyeing sources or for further uses in the following years, it’s sure that it will be planted again by local population or even in industrial scale.

From the literature review and ethnobotanical surveys focused on uses of plants traditionally used in dyeing and conducted from local elderly informants between January 2021 to November 2022, a list of **194** plant species (**72** botanical families, and **157** plant genera) with diverse botanical status (endemic, native, introduced or alien-invasive species) have been inventoried. The entire list of 194 plant species can be found as Additional file 1: Table S1. It demonstrated the potential of these plant species as new sources of water-soluble dyes or pigments, including the endemicity, the IUCN (International Union for Conservation of Nature) and/or the protected status of the species in Reunion Island, the accessibility, the cultivability, the plant organs used for the extraction process, the colors that may be extracted, the scientific knowledge, the industrial interests and current uses of these plant species, amongst others. Dye plants inventoried here include **34** endemic species, **22** native and cryptogenic species, and **138** introduced species (including **37** alien-invasive species) which may have promising properties as new alternative crops for producing bioactive dyes and pigments for the industries.

#### The uses of endemic plant species in Reunion Island potentially rich in dyes or pigments

A total of **13** endemic plant species to Mascarenes, **12** plant species strict endemic species to Reunion Island have been mentioned as having potential traditional uses as sources of dyestuff materials. The botanical information and the coloring properties of all inventoried endemic plant species in Reunion Island potentially rich in dyes or pigments are described in Table 2. Some of them were mentioned in the literature as plants formerly used in textile dyeing, *e.g.*, tanins extracted from wood of *Indigofera ammoxylum* [27] and *Foetidia mauritiana* [28], tanins from barks of *Weinmannia tinctorial* [26, 29, 30]*, Mimusops balata* [26], and *Terminalia bentzoe* subsp. *bentzoe* [26, 30] bluish-brown colors from fruits of *Bertiera borbonica* and *Bertiera rufa* [21], and pigments from stem and leaves of *Psychotria borbonica* [22, 26, 30].

**Table 2:** Endemic plant species in La Réunion Island potentially rich in dyes or pigments

Species	Family	Local vernacular name	Endemicity level	IUCN Red List Categories & Protection status (French law)*	Plant organs	Color produced	Known traditional uses and applications, and cultural status	Main components	Cited references
*Aloe macra* Haw	ASPHODELACEAE	Mazambron marron	Endemic Réunion	EN/Protected	Leaves	Dark yellow; green	Ornamental, medicinal uses; cultivated for conservation purposes	Anthraquinones, flavonoids	[38]
*Bertiera borbonica* A. Rich. ex DC	RUBIACEAE	Bois de raisin	Endemic Réunion	DD	Fruits	Bluish, brown	Medicinal uses; cultivated occasionally	Alkaloids, flavonoids, saponosids, tannins, leucoanthocyans	[21]
*Bertiera rufa* DC	RUBIACEAE	Bois de raisin	Endemic Réunion	LC	Fruits	Bluish, brown	Medicinal uses; cultivated occasionally	Alcaloids, flavonoids, saponosids, tannins, leucoanthocyans	[21]
*Erythroxylum laurifolium* Lam	ERYTHROXYLACEAE	Bois de rongue	Endemic Réunion-Mauritius	LC	Bark, stems	Red	Medicinal uses,; cultivated occasionally	Proanthocyanidins, condensed tannins, flavonoids (quercitrin and afzelin)	[39, 40]
*Ficus densifolia* Miq	MORACEAE	Affouche	Endemic Réunion-Mauritius	LC	Wood	Red	Cultivated occasionally	n.d	*–*
*Foetidia mauritiana* Lam	LECYTHIDACEAE	Bois puant	Endemic Réunion-Mauritius	CR/Protected	Wood	Red-brown	Ornamental; cultivated for ornament and conservation purposes	Tannins	[18, 28, 41]
*Forgesia racemosa* J.F. Gmel	ESCALLONIACEAE	Bois de Laurent-Martin	Endemic Réunion	LC	Stems	n.d	Ornamental; uncontrolled culture	n.d	*–*
*Hubertia ambavilla* Bory var.* ambavilla*	ASTERACEAE	Ambaville	Endemic Réunion	LC	Flower	Yellow-green	Medicinal uses, ornamental; cultivated for economic purposes	n.d	*–*
*Hypericum lanceolatum* Lam	HYPERICACEAE	Fleur jaune	Endemic Réunion-Comoros	LC	Flower	Bright yellow	Medicinal uses, ornamental; cultivated for economic purposes	n.d	*-*
*Indigofera ammoxylum* (DC.) Polhill	FABACEAE	Bois de sable	Endemic Réunion	CR/Protected	Wood	Red	Ornamental, wood stain; cultivated for ornament and conservation purposes	Tannins	[16, 18, 27]
*Latania lontaroides* (Gaertn.) H.E. Moore	ARECACEAE	Latanier rouge	Endemic Réunion	CR/Protected	Leaves	Black	Ornamental; cultivated for ornament and landscaping	Alkaloids	*–*
*Mimusops balata* (Aubl.) C.F. Gaertn	SAPOTACEAE	Grand natte	Endemic Réunion-Mauritius	LC	Bark	Brown	Ornamental; cultivated for forestry purposes	Tannins	*–*
*Molinaea alternifolia* Wild	SAPINDACEAE	Tan Georges	Endemic Réunion-Mauritius	LC	Bark, stems	Brown	Medicinal uses; cultivated occasionally	n.d	[21, 26]
*Monimia ovalifolia* Thouars	MONIMIACEAE	Mapou à petites feuilles	Endemic Réunion-Mauritius	LC	Bark	n.d	-; uncontrolled culture	Tannins	*–*
*Ochrosia borbonica* J.F. Gmel	APOCYNACEAE	Bois jaune	Endemic Réunion-Mauritius	VU/Protected	Bark	Yellow	Medicinal uses; cultivated for conservation purposes	Alkaloids	[34–37]
*Olax psittacorum* (Lam.) Vahl	OLACACEAE	Corce rouge, bois d'effort	Endemic Réunion-Mauritius	VU	Bark	Red, orangish	Medicinal uses;; cultivated occasionally	n.d	[42]
*Psiadia anchusifolia* (Poir.) Cordem	ASTERACEAE	Bouillon blanc	Endemic Réunion	LC	Whole plant	Greenish-brown	Medicinal uses; uncontrolled culture	Flavonoids	[43, 44]
*Psiadia callocephala* (Bory) Cordem	ASTERACEAE	-	Endemic Réunion	LC	Whole plant	Greenish-brown	Medicinal uses; uncontrolled culture	Flavonoids	[43, 44]
*Psiadia dentata* (Cass.) DC	ASTERACEAE	Ti mangue	Endemic Réunion	LC	Whole plant	Greenish-brown	Medicinal uses; cultivated occasionally	Flavonoids	[43, 44]
*Psychotria borbonica* (J.F. Gmel.) Razafim. B.Bremer	RUBIACEAE	Bois cassant	Endemic Réunion-Mauritius	LC	Stems, leaves	n.d	Medicinal uses; uncontrolled culture	Alcaloids, flavonoids, saponosids, tannins, leucoanthocyans	[22]
*Ruizia cordata* Cav	MALVACEAE	Bois de senteur blanc	Endemic Réunion	CR/Protected	Bark	n.d	Ornamental; cultivated for ornament and conservation purposes	n.d	*–*
*Sophora denudata* Bory	FABACEAE	Petit tamarin des Hauts	Endemic Réunion	EN/Protected	Bark, leaves	n.d	Softwood lumber; medicinal uses; cultivated occasionally	Flavonoids	[45, 46]
*Terminalia bentzoe* (L.) L.f. subsp.* bentzoe*	COMBRETACEAE	Benjoin	Endemic Réunion-Mauritius	CR/Protected	Bark	Yellow, black	Medicinal uses, ornamental; cultivated for ornament and landscaping	Tannins	[18, 26, 30, 32, 47]
*Weinmannia mauritiana* D. Don	CUNONIACEAE	Petit bois de tan	Endemic Réunion-Mauritius	LC	Bark	n.d	Uncontrolled culture	Tannins	*–*
*Weinmannia tinctoria* Sm	CUNONIACEAE	Tan rouge	Endemic Réunion-Mauritius	LC	Bark	Red	Leather dyeing (not today); cultivated today to support honey production in Reunion Island; cultivated for ornament and landscaping	Tannins	[17, 29, 30]

*NE* Not Evaluated, *DD* Data Deficient, *LC* Least Concern, *NT* Near Threatened, *VU* Vulnerable, *EN* Endangered, *CR* Critically Endangered, *EW* Extinct in the Wild and *EX*, *n.d.* not determined

*IUCN Red List Categories

On the other hand, the traditional uses of some endemic plants as textile dyestuffs have only been identified from the ethnobotanical survey conducted here, such as the uses of bark of *Ochrosia borbonica* (locally called “bois jaune”—yellow wood), the barks and stems of *Erythroxylum laurifolium*, the flowers of *Hypericum lanceolatum*, the leaves of *Latania lontaroides*, the bark of *Monimia ovalifolia*, and *Sophora denudata*, amongst others.

Some of these endemic species are classified by the International Union for Conservation of Nature (IUCN) as Vulnerable (VU), Endangered (EN), or Critically Endangered (CR). For example, eight endemic species inventoried here are protected in Reunion Island by local environmental legislation, *e.g.*, *Indigofera ammoxylum* (CR), *Foetidia mauritiana* (CR), *Ruizia cordata* (CR), *Latania lontaroides* (CR), *Terminalia bentzoe* subsp. *bentzoe* (CR), *Aloe macra* (EN), *Sophora denudata* (EN), and *Ochrosia borbonica* (VU). Interestingly, *L. lontaroides* and *T. bentzoe* subsp. *Bentzoe—*both classified as CR by IUCN—are two species widely available and easily cultivated in Reunion Island. Thus, they may have promising interests as local sources of natural dyes or pigments for the industries. Edible fruits of *L. lontaroides* contain oil seeds with very similar composition to that of the edible palm oil [31] but the coloring properties of the species have never been described in the literature. Barks of *T. bentzoe* subsp. *bentzoe* have interesting biological properties like antioxidant and cytotoxic activities [32]. The barks of this plant are rich in tannins and can be used to dye natural fibers with a black or yellow color, depending on the mordant used during natural dyeing [18, 26, 30, 33].

On the other hand, the bark of the endemic species *O. borbonica* has been exploited without a sustainable policy and almost led to the extinction of this species in the Mascarenes. This plant species has been used by local population for its medicinal properties against malaria and fever many years ago. Only a few trees have been recently inventoried in Reunion Island and the species are locally protected. The health benefits of *O. borbonica* have been proven by the most recent pharmacological research [34–37]. Thus, the bark of *O. borbonica* can produce a yellow dyestuff rich in bioactive alkaloids with therapeutic activities. It would be interesting to relaunch the study on public and/or private partnership reforestation projects with this promising but endangered plant, *O. borbonica.* It may led to the conservation of this highly-valued species rich in bioactive yellow dyes.

Furthermore, even if the species *W. tinctoria* is classified as endangered in some parts of the world by IUCN, this tree is not locally protected and very common in Reunion Island (in the wild and private gardens). It is used as an ornamental plant. The plantation of this species has resumed recently to support the specific honey production from Reunion Island called “miel vert” (green honey). The bark of *W. tinctoria* has been used for its dyeing properties to dye leather in red color a long time ago. It is a source of tannins [29]. However, very limited information is available on the coloring properties and dye composition of the dyestuffs extracted from bark of *W. tinctoria*, and it may become an important and interesting topic for future research on natural dyeing from tropical dye plants.

Finally, other recorded endemic species, not protected and widely available in the island (Table 2), such as fruits of *Olax psittacorum* (locally called “corce rouge”)*, Bertiera rufa, Erythroxylum laurifolium, Hypericum lanceolatum, Monimia ovalifolia, Weinmannia mauritiana,* etc., also represent an interesting group of alternative crops for natural dyeing application, if they will be exploited in a sustainable way by local population.

#### The uses of native plant species in Reunion Island potentially rich in dyes or pigments

All the inventoried native and cryptogenic plant species in Reunion Island potentially rich in dyes or pigments are described in Table 3. Among the **23** species listed below, many of them are also growing in Madagascar [6, 9, 24] and inventoried as dye-producing plants, as the example of *Thespesia populnea Danais fragrans* [9], and *Morinda citrifolia* [48–50]*.* The cryptogenic species *T. populnea* (Malvaceae; locally called ‘Porché’) is widely and easily cultivated in Reunion Island, and should be used as an efficient alternative source of reddish natural dyestuffs [6, 51–54]. *D. fragrans* (locally called ‘Liane jaune’—yellow liana) and *M. citrifolia* (noni, or locally called ‘malaye’) are native plant species from the Rubiaceae family. The uses of roots as sources of anthraquinonoid dyes are common in this botanical family (like the case of the famous *Rubia tinctorum*) [55]. The species *M. citrifolia* is widely available and easily cultivated in Reunion Island, whereas *D. fragans* is a relatively rare species in the wild, but the roots of these two Rubiaceae plants could be used for natural dyeing properties (yellowish to orangish-red) [6, 49].

**Table 3:** Native and cryptogenic plant species in La Réunion Island potentially rich in dyes or pigments

Species	Family	Local vernacular name	Endemicity level	IUCN Red List Categories & Protection status (French law)*	Plant organs	Color produced	Known traditional uses and applications, and cultural status	Main components	Cited references
*Agarista salicifolia* (Comm. ex Lam.) G. Don	ERICACEAE	Bois de rempart	Native (Africa, Madag., Réunion, Mauritius);	LC	n.d	n.d	Ornamental; cultivated occasionally	n.d	*–*
*Alectra sessiliflora* (Vahl) Kuntze	OLEACEAE	-	Native (E. Africa, Madag. to S. China and Philippines)	LC	Fowers, roots	Yellow	Medicinal uses; uncultivated	n.d	[57]
*Antidesma madagascariense* Lam	PHYLLANTHACEAE	Bois de cabri blanc	Native (Madag., Comoros);	LC	n.d	n.d	Ornamental; cultivated occasionally	n.d	*–*
*Antirhea borbonica* J.F. Gmel	RUBIACEAE	Bois d’osto	Native (Madagascar, Mauritius);	LC	Leaves, bark	Greenish	Medicinal uses; cultivated occasionally	alcaloids, flavonoids, phenols, triterpens, tetrapyrrols	[21, 58–60]
*Aphloia theiformis* (Vahl) Benn	APHLOIACEAE	Change-écorce	Native (SE trop. Africa, Madag., Comoros, Mascarenes, Seychelles);	LC	Leaves, stem, bark	Yellow	Medicinal uses; cultivated occasionally	Flavonoids, tannins, Mangiferine	[6, 24]
*Cassytha filiformis* L	LAURACEAE	Liane foutafout	Native (Pantropical); invasive	LC	Liana	Orangish, yellow	Medicinal uses; uncultivated	Alkaloids, flavonoids	[6, 61, 62]
*Crotalaria retusa* L	FABACEAE	Pois rond marron, crotalaire	Cryptogenic (Pantropical)	NA	n.d	n.d	Toxic plants; uncultivated	Alcaloïds (pyrrolizidine, monocrotaline, retrorsine)	[22, 63]
*Danais fragrans* (Lam.) Pers	RUBIACEAE	Liane jaune	Native (Mauritius, Madag.);	LC	Roots	Orangish-red	Ornamental, medicinal uses; cultivated occasionally	Quinonoid	[9, 29]
*Dianella ensifolia* (L.) DC	ASPHODELACEAE	Vacoa nain, reine des bois	Cryptogenic (Madag., Seychelles, Asia);	DD	Fruits	Blue	Medicinal uses; cultivated for ornament	Anthocyanins	[64, 65]
*Dodonaea viscosa* Jacq	SAPINDACEAE	Bois d’arnette	Native (Pantropical);	LC	Leaves, bark	Yellow, dark	Ornamental, medicinal uses; cultivated for ornament and landscaping	n. d	*–*
*Doratoxylon apetalum* (poir.) Radlk	SAPINDACEAE	Bois de gaulette	Native (Madag., Mascarenes)	LC	Leaves	n.d	Ornamental; cultivated occasionally	n. d	[59]
*Ficus rubra* Vahl	MORACEAE	Affouche rouge	Native (Madag., Comoros, Seych., Mascarenes);	LC	Wood	Red	Ornamental; uncultivated	n. d	*–*
*Leea guineensis* G. Don	VITACEAE	Bois de sureau	Native (Trop. Africa, Madag., Mauritius)	LC	Barks	Red	Ornament, medicinal uses; cultivated for ornament and landscaping	Flavonoids, tannins, terpenes	[6]
*Morinda citrifolia* L	RUBIACEAE	Malaye, noni	Native (Indopacific)	DD	Roots	Orange, Yellow	Food and medicinal uses; cultivated occasionally	Polyphenol, flavonoid, quinonoid	[48–50]
*Mussaenda arcuata* Poir	RUBIACEAE	Lingue café	Native (Mascarenes, Madag., Africa);	LC	Roots, flowers	Orangish-red	Medicinal uses; cultivated occasionally	n. d	[6, 66]
*Olea lancea* Lam	OLEACEAE	Bois d’olive blanc	Native (Madag., Mascarenes)	LC	Leaves	n.d	Ornamental; cultivated for ornament and landscaping	n. d	*–*
*Pandanus utilis* Bory	PANDANACEAE	Vacoa	Native (Mascarenes)	LC	Terminal buds, leaves	Yellow, green	Ornamental, food uses; Cultivated for economic purposes and for landscaping	n. d	[56]
*Pemphis acidula* J.R. Forst. et G. Forst	LYTHRACEAE	Bois matelot	Native (Paleotropical);	LC	Barks	Red	Ornamental; cultivated for conservation purposes	n. d	*–*
*Phymatosorus scolopendria* (Brum f.) Pic Serm	POLYPODIACEAE	Patte-lézard	Native (Africa, trop. Asia to Austr. and Polynesia);	LC	Leaves	Greenish	Medicinal uses; cultivated occasionally	Saponines	[25]
*Pteridium aquilinum* (L.) Kuhn	DENNSTAEDTIACEAE	Fougère aigle	Native (Cosmop.); invasive	LC	Leaves, fruits	Black	, medicinal uses; uncultivated	Flavonoids, tannins, alkaloids	[67]
*Securinega durissima* J.F. Gmel	PHYLLANTHACEAE	Bois dur	Native (Mascar., Madag., Mayotte);	LC	Bark, sap	Reddish	Ornamental, medicinal uses; cultivated for ornament and landscaping	n. d	[46]
*Talipariti tiliaceum* (L.) Fryxell	MALVACEAE	Mova, var	Native (Trop. and subtrop. regional coast) cultivated	EN/Protected	Bark, leaves	n.d	Ornamental, medicinal uses; cultivated for ornament and landscaping	Quinones, lapachol	*–*
*Thespesia populnea* (L.) Sol. Ex Corrêa	MALVACEAE	Porché	Cryptogenic (Coastal pantropical) cultivated	DD	Fruits, flowers, bark	Red to light brown	Medicinal uses; cultivated for ornament and landscaping	Polyphenols, flavonoids, tannins, terpenes, alkaloids	[6, 51, 52, 54, 68]

*NE* Not Evaluated, *DD* Data Deficient, *LC* Least Concern, *NT* Near Threatened, *VU* Vulnerable, *EN* Endangered, *CR* Critically Endangered, *EW* Extinct in the Wild and *EX*, *n.d.* not determined

*IUCN Red List Categories

The native species *Leea guineensis* (Vitaceae; locally called ‘Bois de sureau’) is a tree that is very common in wetlands in Reunion. Its rapid growth and strong regeneration capacities make it an invasive plant in the wild. The barks of this species are rich in flavonoids, tannins and terpenes [6], and they could produce red dyes with tinctorial property. The same applies to terminal bud and leaves of the native cultivated species *Pandanus utilis* (Pandanaceae; locally called ‘Vacoa’) which contain bioactive components [56] and could produce yellowish and greenish textiles dyes according the ethnobotanical data recorded here.

Furthermore, other inventoried native species (today not cultivated in Reunion, but easily accessible in wilderness area and not protected) should also be promising for natural dyeing application, such as liana of *Cassytha filiformis* (Lauraceae) for orangish-yellow colors; flowers and roots of *Mussaenda arcuata* (Rubiaceae) for orangish-red shades; barks of *Pemphis acidula* (Lythraceae) and *Securinega durissima* (Phyllanthaceae) for reddish shades; barks of *Antirhea borbonica* (Rubiaceae), leaves of and *Phymatosorus scolopendria* (Polypodiaceae) for greenish colors; or leaves and fruits of *Pteridium aquilinum* (Dennstaedtiaceae) for black shades. However, additional studies are necessary to confirm the pigment properties and tinctorial strength on natural fabrics of these uncharacterized tropical plants.

#### Introduced and alien-invasive species in Reunion Island potentially rich in dyes or pigments

Actually, Reunion Island contains several microclimates where the pedological properties and the climates vary from one area to one another. It explains that a multitude of species and botanical family of plants with well-known dyeing properties are able to acclimatize to this island. Nevertheless, unlike the history behind dye plants sectors in Madagascar, this field of natural colorants and pigments have never been really developed in Reunion island. Traces of industrial plantation and uses of *Indigofera tinctoria* have been quoted during the ethnobotanical survey in the north of the island. Unfortunately, it has been replaced by other crops because at that time, natural colorants didn’t have the current importance in the world market. **138** introduced species potentially rich in dyes or pigments have been listed, and most of them are cultivated in the island (see Table 4). Most relevant examples of the introduced cultivated plants rich in dyes or pigments are *Curcuma longa* [69, 70], *Selenicereus undatus* (syn. *Hylocereus undatus*; called ‘Pitahaya’) [71], *Lawsonia inermis* [72], *Terminalia catappa* [73, 74], *Haematoxylum campechianum* [75], and *Casuarina equisetifolia* [76, 77], amongst others. The species *C. longa* and *S. undatus* are widely cultivated and used in the island for local foods [69, 70]. Interestingly, according to the ethnobotanical data recorded here, a large number of alien-invasive plants in Reunion are potentially rich in dyes or pigments, such as *Leucaena leucocephala* [78]*, Antigonum leptopus* [79], *Acacia dealbata* [80], *Casuarina equisetifolia* [77], etc.

**Table 4:** Examples of alien cultivated and invasive plant species in La Reunion Island potentially rich in dyes or pigments

	Family	Local vernacular name	General status (region of origin)	IUCN Red List Categories & Protection status (French law)	Plant organs	Color produced	Known traditional uses and applications, and cultural status	Main components	Cited references
*Acacia dealbata* Link	FABACEAE	Acacia Bernier, mimosa	Alien invasive (Australia, Tasmania)	NA	Bark, pods, flowers	Black	Medicinal uses; restoration; horticulture	Flavonoid, alkaloid, tannins	[80, 81]
*Acacia mearnsii* De Wild	FABACEAE	Acacia	Alien invasive (Australia)	NA	Bark, pods	Yellow, pink, grey, brown, black	Medicinal uses; horticulture; firewood	Tannins, proanthocyanidin	[81, 82]
*Antigonon leptopus* Hook. et Arn	POLYGONACEAE	Liane antigone	Alien invasive (Mexico and Central America)	NA	Leaves, stems and flowers	Brown, green	Medicinal uses; ornament	Phenolic compounds, tannins, anthraquinons	[79]
*Casuarina equisetifolia* L	CASUARINACEAE	Filao	Alien cultivated, invasive (Indopacific litt., Australia and New-Caledonia)	NA	Bark, leaves	Pink, brown	Dyeing; firewood; against beach erosion	Casuarine	[76, 77]
*Cinnamomum burmannii* (Nees et T. Nees) Blume	LAURACEAE	Ti cannelle	Alien cultivated, invasive (South-East Asia)	NA	Leaves	Red	Medicinal uses; dietary	D-Borneol	[83]
*Coccoloba uvifera* (L.) L	POLYGONACEAE	Raisin de mer	Alien cultivated (Atlantic American littoral)	NA	Fruit, leaves	Purpleish	Ornament; medicinal uses; dietary; woodworking	Anthocyanins, anthocyanidins,	[84]
*Cryptomeria japonica* (L. f.) D. Don	CUPRESSACEAE	Cryptoméria	Alien cultivated (Japan)	NA	Leaves	Copper apricot, flesh color	Medicinal uses; *essential oil; insecticide;* woodworking	Terpinene, p-cymene, 3-carene, terpinolene, beta-myrcene	/
*Curcuma longa* L	ZINGIBERACEAE	Safran-pays	Alien cultivated (India)	NA	Rhizome	Dark yellow	Medicinal uses; dietary	Polyphenols, curcumins	[85, 86]
*Leucaena leucocephala* (Lam.) de Wit	FABACEAE	Cassi	Alien invasive (Central America)	NA	Leaves	n.d	Medicinal uses; fodder; firewood	Tannins	[87]
*Phormium tenax* J.R. Forst. et G. Forst	ASPHODELACEAE	Vacoa de laine	Alien cultivated (New Zealand)	NA	Whole plant	Yellow	Ornament; textile	n.d	/
*Psidium cattleyanum* Sabine	MYRTACEAE	Goyavier	Alien cultivated and invasive (Brazil)	NA	Wood, fruit	Reddish	Medicinal uses; uncontrolled culture; dietary; woodworking	Anthocyanins, Phenolics, carotenoids	[88]
*Ravenala madagascariensis* Sonn	STELITZIACEAE	Ravenale, arbre du voyageur	Alien cultivated, naturalized (Madagascar)	NA	Fruit	Turquoise	Ornament	n.d	/
*Selenicereus undatus* (Haw.) D.R. Hunt	CACTACEAE	Pitahaya	Alien cultivated, invasive (Tropical America)	NA	Fruit	Red, purple	Medicinal uses; dietary; defensive hedges	n.d	[71]
*Syzygium cumini* (L.) Skeels	MYRTACEAE	Jamblon	Alien invasive, cultivated (Indo-Malaisia)	NA	Fruit	Purple, yellowish	Medicinal uses; uncontrolled culture; dietary	Tannins	[89]
*Terminalia catappa* L	COMBRETACEAE	Badamier	Alien (cryptogenic?) cultivated (Indopacific litt.)	NA	Leaves, bark	Brownish	Medicinal uses; dietary; ornament (shady); firewood; woodworking	Tannins	[6, 73, 74]
*Ulex europaeus* L	FABACEAE	Ajonc d'Europe, zépinard des Hauts, Genêt	Alien invasive (Europe)	NA	Flowers	Pale yellow	Defensive hedges;fodder	n.d	[9]

### First selection and characterization of some promising dye plants in Reunion Island

Based on different criteria, like endemicity, scientific knowledge, accessibility and availability, cultivability, plant organs used for the extraction, and the industrial interests and other known applications of the species, we established a classification method to finally select the most interesting and widely available dye plant species listed above. From this first collection of Reunion Island’s dye plants, we valued endemic and native dye plants because of their value to the extension and conservation of the Island plant biodiversity, and also for their potential as a locally available resource that can lead to regional economic development. Thus, **15** endemic and **13** native species (*i.e.*, **58%** of the endemic and native species inventoried below) have been harvested from different growing areas in Reunion Island. The results of our laboratory experiments in terms of extraction yield and coloring properties (shade, solubility and stability) of the colors that have been extracted from each dye plant harvested, as ranked by their ‘dye score,’ are described in Table 5. Some colors are more interesting than others in terms of industrial dyeing applications, thus we valued rare/special colors (*e.g.*, blue, magenta, pink, cyan, mauve, black…) as well as yellow, orange and red shades which are sought in the dyeing industries to replace synthetic azo dyes. Moreover, the colors extraction yield from the plant organs, and the capacity to obtain stable water-soluble dyes extracted easily and efficiently using eco-extraction method at low temperature with water or aqueous ethanolic solution as solvents are also two main factors contributing to the success of the industrial use of the considered plant-based dyestuffs.

**Table 5:**
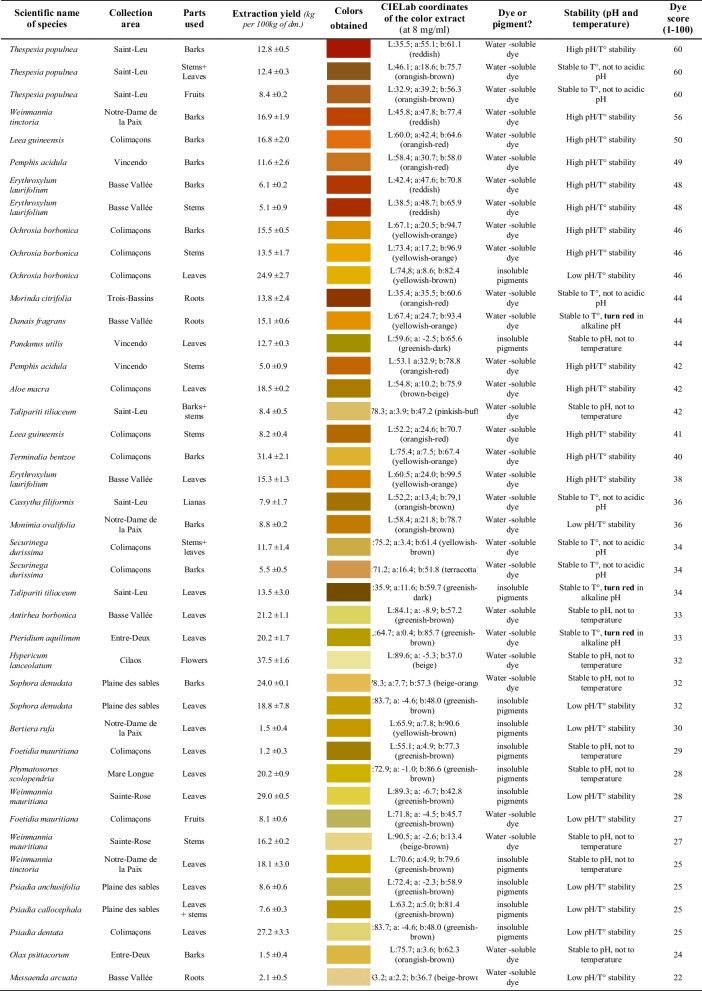
Colors obtained from endemic and native dye plants in Reunion island as ranked by their dye score

The species *Thespesia populnea* sets itself apart by its high dye score of 60 (from a scale of 1 to 100). This is the highest dye score obtained among all the endemic and native plant species inventoried in this study (Table 5). First, a value of 9 was attributed to the endemicity index (criteria C1*C2; Tab.1) considering that *T. populnea* is a cryptogenic species in Reunion and few information is available on the coloring properties of the flowers and fruits of this species [51, 54, 68]. However, only little information is available on the dyeing properties of the barks analyzed here, and the nature of the pigments from the red barks is not established. The cultivability index (criteria C3*C4; Tab.1) was scored at 100 because *T. populnea* is easily cultivated in the island, and the industrial extrapolation index (criteria C5*C6; Tab.1) presents the value of 49 because the barks are used to extract the dyes, and this plant species has other industrial interests in Reunion island (*e.g*., medicinal uses). Then, the coloring strength index, based on the criteria C7 (color and stability of dyes and pigments extracted) and C8 (yield and difficulty of color extraction), is the highest among all the plants investigated here. Indeed, *T. populnea* is characterized by the reddish bloody color of its bark extract (with the highest red a*-value of + 55.1 in the CIELab system among all the color extracts analyzed) with a promising extraction yield of 12.8 kg per 100 kg of barks (dry matter; dm.) obtained after a solid/liquid eco-extraction using ethanol and water mixture (70:30, v/v), and a high pH and temperature stability of the extract containing reddish water-soluble dyes (Table 5).

Red color is one the most used color in the industries. Even if sources of reddish colors are already available in the market like carminic acid from *Dactylopius coccus* or bixin from *Bixa orellana*, the research of other natural sources are still in demand because the actual colorants in the market don’t really offer the bright red color that their artificial counterparts offer. Thus, our results demonstrated that barks of *T. populnea*, as well as the dyestuff extracted from barks of *Weinmannia tinctoria* (a*-value of + 47.8; yield of 16.9 kg per 100 kg dm.; dye score of 56), barks of *Leea guinensis* (a*-value of + 42.4; yield of 16.8 kg per 100 kg dm.; dye score of 50), barks of *Pemphis acidula* (a*-value of + 30.7; yield of 11.6 kg per 100 kg dm.; dye score of 49), barks of *Erythroxylum laurifolium* (a*-value of + 48.7; yield of 6.1 kg per 100 kg dm.; dye score of 48). Roots of *Morinda citrifolia* (orangish-red color; a*-value of + 35.5; yield of 13.8 kg per 100 kg dm.; dye score of 44) offered promising color extracts containing water-soluble dyes which give a bright red or orangish-red coloration at low concentrations (from 2 to 8 mg/mL) (Table 5). The wavelength of maximum absorbance obtained around 500 and 550 nm in visible confirms the reddish color given by these selected plant-based dyestuffs. These red colors may come from different chemical classes of natural dyes like tannins, proanthocyanidins, flavonoids, alkaloids, anthraquinones, etc. (Tables 2, 3). Furthermore, the reddish colors of *T. populnea*, *W. tinctoria*, *L. guinensis, P. acidula, E. laurifolium* and *M. citrifolia* extracts can be considered as highly stable considering that they are able to support a wide range of pH (from 4 to 10 in water buffer solutions) and temperature (from 25 to 95 °C) with no significant variation; only an instability (discoloration) to acidic pH was observed for the extract of *M. citrifolia* roots.

Natural source of orangish and yellowish colorants are the second most used colorants in the dye industries. With red and blue, yellow is classified as one of the primary colors. Even if many sources of yellow colors are proposed in the market like curcumin or carotenoids (lutein…), the main limits of these colorants are their low stability. Our results indicated that many dye plant species from Reunion Island contain orangish and yellowish dyes. These colored molecules can be alkaloids, flavonoids, quinonoids, etc. Three plant-based dyestuffs, namely barks and stems of *Ochrosia borbonica* (b*-value of + 94.7; yield of 15.5 kg per 100 kg dm.; dye score of 46), roots of *Danais fragrans* (b*-value of + 93.4; yield of 15.1 kg per 100 kg dm.; dye score of 44), and barks of *Terminalia bentzoe* (b*-value of + 67.4; yield of 31.4 kg per 100 kg dm.; dye score of 40) give a bright yellowish-orange color (yellow at low concentration and orange in concentration of 8 mg/ml or greater) (Table 5). Stability characterization performed on these color extracts demonstrates a high stability toward temperature and pH of *O. borbonica* and *T. bentzoe* extracts, and a high stability specially toward temperature for *D. fragrans* extract; interestingly, this last extract turns red in alkaline pH (Table 5). Compared to commercial *Reseda luteola* extract, a common dye plants used in European country as a yellow colorant, the color stability of these above interesting sources of yellowish-orange colorants from Reunion Island is very promising for dyeing applications. This current study offered a real choice of new natural sources of yellowish-orange dyes that remain stables.

Then, greenish-brown, orangish-brown, yellowish-brown, beige, or pale beige-orange colors were obtained by the others endemic and native species harvested in this study (Table 5). For instance, the leaves of *Pandanus utilis* (yield of 12.7 kg per 100 kg dm.; dye score of 44) produce a pale greenish extract at low concentration, but give a bright greenish-dark color at a concentration of 8 mg/ml. The color remains greenish a few days later both in acidic and alkaline medium. This property is very uncommon for pigments extracted from green leaves and it will be of interest to analyze the chemical classes of the pigments extracted. The leaves of *Talipariti tiliaceum* (yield of 13.5 kg per 100 kg dm.; dye score of 34) also give a greenish-dark color extract of interest for dyeing application. Interestingly this greenish-brown color turns red in alkaline pH, and the same applies to leaves of the species *Pteridium aquilinum* which give an intense red in alkaline pH. Furthermore, from a mixture of barks and stems of the native species *T. tiliaceum*, an original pinkish-buff shade was obtained.

Finally, a selection of **15** introduced cultivated or alien-invasive species in Reunion Island, among the 138 alien species inventoried below (Table 3 and Additional file 1: Table S1), have also been harvested and characterized in this part of the study. Main results in terms of extraction yield and coloring properties (shade, solubility and stability) for these introduced species are summarized in Table 6. Water-soluble dyes with various shades were obtained according to the dye plants, like pink-violet from fruits of *Selenicereus undatus* (cultivated), yellowish-orange shade for rhizomes of *Curcuma longa* (cultivated), leaves of *Terminalia catappa* (cultivated), stems of *Antigonum leptopus* (invasive), barks of *Casuarina equisetifolia* (invasive) or fruits of *Coccoloba uvifera* (cultivated), and greenish shade for leaves of *Leucena leucocephala* (invasive). The Reunion’s forest is a remarkable reserve of plant biodiversity, and the most promising dye plant species (endemic, native, alien cultivated or invasive) may be used in dyeing application for the industries subject to their successful application for coloring textiles, food or cosmetics.

**Table 6:**
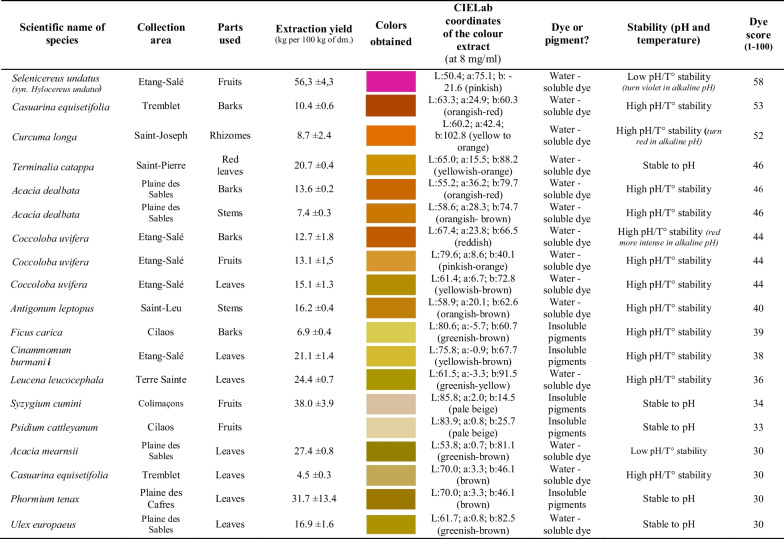
Colors obtained from some alien cultivated or invasive dye plants in Reunion island (as ranked by their dye score)

### Bath-dyeing applications on natural fibers of dye plants from Reunion Island

**Fig. 2:**
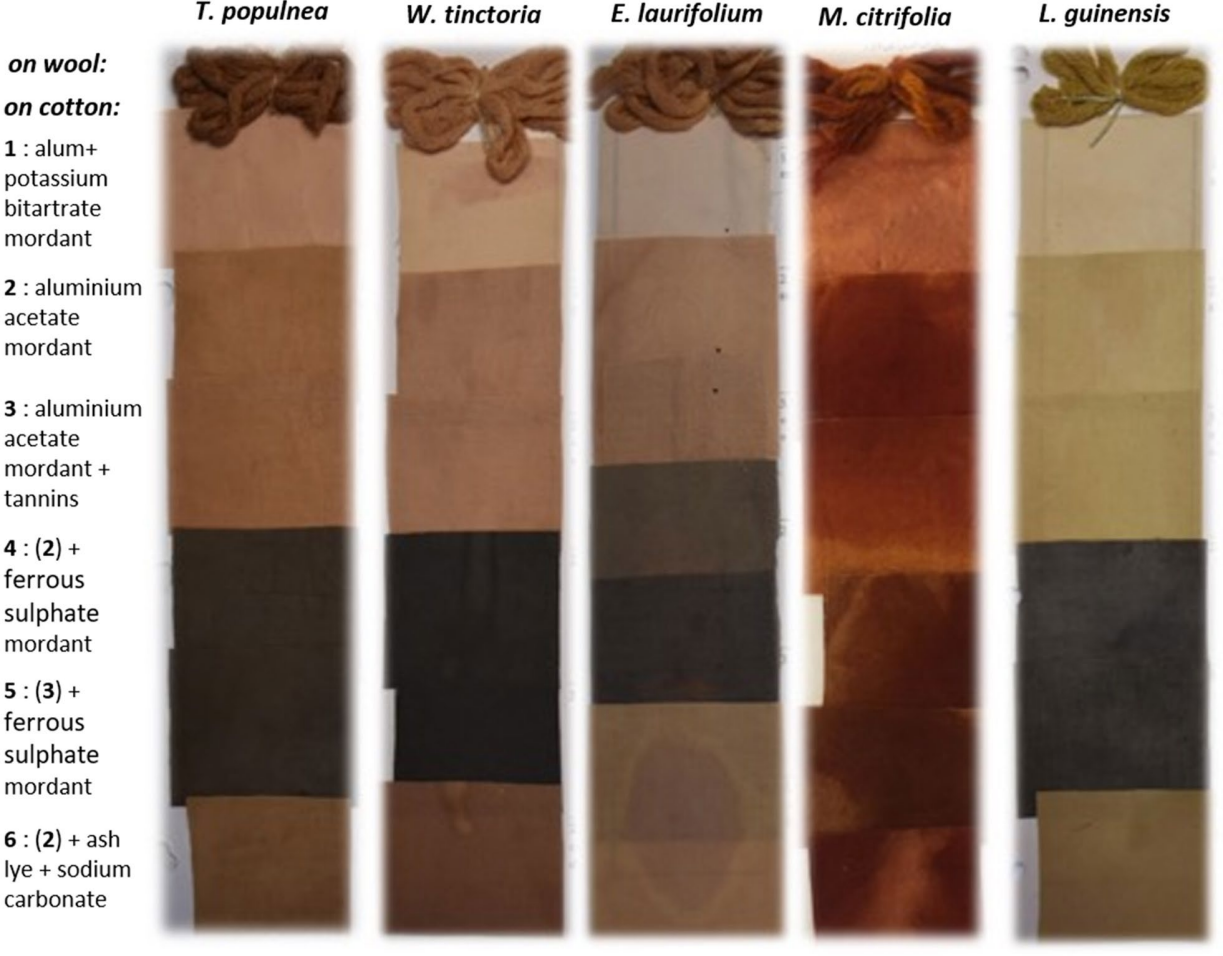
Natural wool and cotton dyeing by bath-dyeing process utilizing the reddish color extracts from *T. populnea, W. tinctoria, E. laurifolium, M. citrifolia,* and *L. guinensis. Six different mordants were added in the dye-bath (1–6)*

**Fig. 3:**
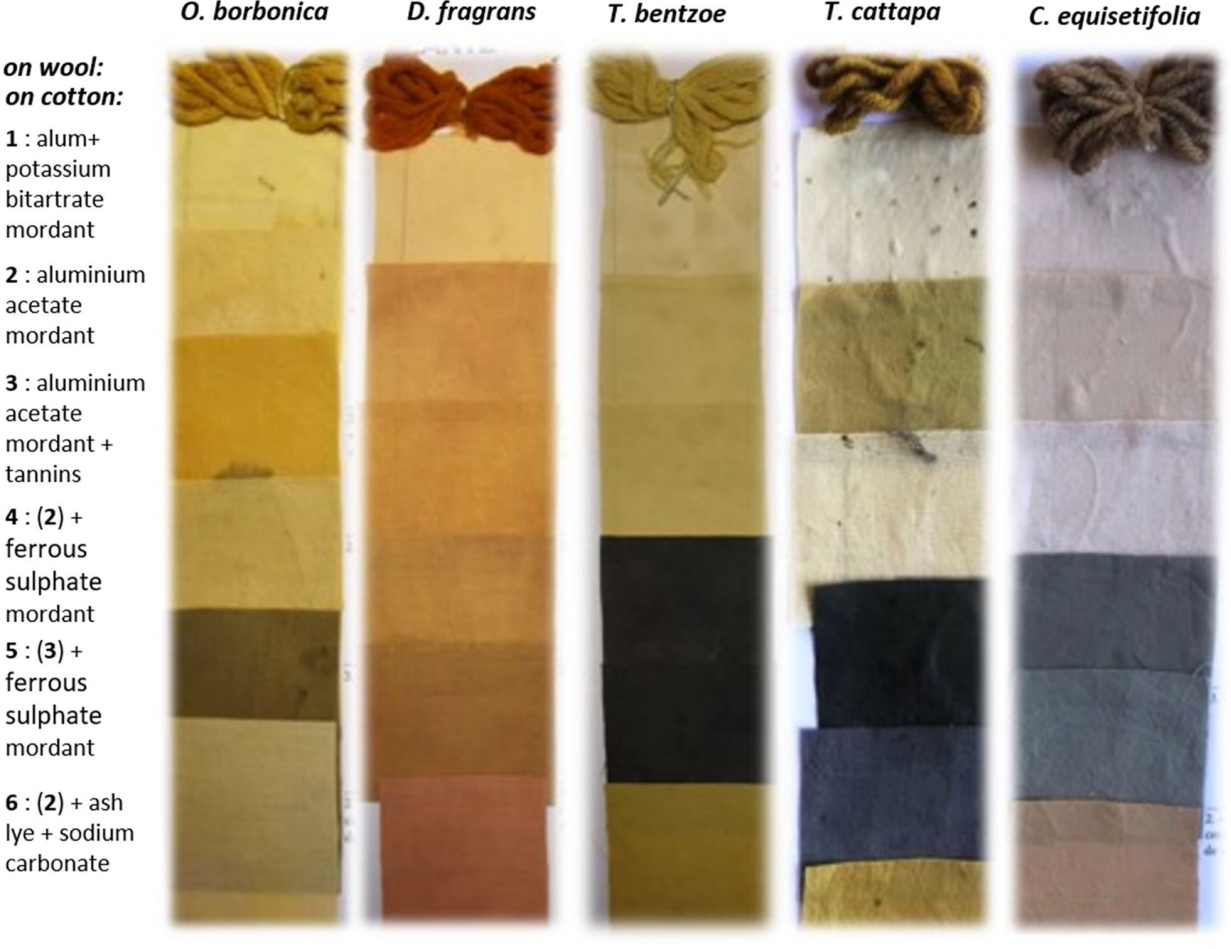
Natural wool and cotton dyeing by bath-dyeing process utilizing the yellowish-orange color extracts from *O. borbonica, D. fragrans, T. bentzoe, T. cattapa*, and *C. equisetifolia*. *Six different mordants were added in the dye-bath (1–6)*

Textile industry is a huge industry and this is crucial to change the way textiles are designed to an eco-friendlier production by using natural textile dyes [10]. Proven toxicity and environmental burdens caused by artificial azo dyes and related hazardous substances applied for textile dyeing, especially in Asia–Pacific region (China and India), have motivated consumers and industrials to turn to natural and eco-friendly fabric dyeing alternatives [2]. In this study, for the first time to our knowledge, the most promising plant-based dyestuffs from Reunion island rich in water-soluble dyes have been investigated for textile dyeing application on natural fibers (wool and cotton). For cotton dyeing, it is usually necessary to perform a mordanting step to improve the dye strength fixation on the fabrics. Choice of mordant is important insofar each complex mordant-textile may give a specific color and light. Thus, the dyeing property of the plant extracts has been checked for bath-dyeing with different mordants (alum, aluminum acetate, tannins, and ferrous sulphate, either alone or in combination). Main results and shades obtained after the bath-dyeing process using the reddish color extracts from *T. populnea, W. tinctoria, E. laurifolium, M. citrifolia*, and *L. guinensis* are presented in Fig. 2. Similarly, results obtained after the bath-dyeing process utilizing the yellowish-orange color extracts from *O. borbonica, D. fragrans, T. bentzoe, T. cattapa,* and *C. equisetifolia* are described in Fig. 3. These experiments have demonstrated very successful and very positive results. By using the adequate dye plant and mordant, it is possible to color the fabrics in a variety of different colors, like bright red with *M. citrifolia* extract, reddish with *W. tinctoria*, terracotta and pinkish-orange color with *T. populnea* and *E. laurifolium* (turn dark-green with iron mordant), sandy (pale yellow, cream) color with *L. guinensis*, bright yellowish-khaki color with *O. borbonica* (turn orangish pink with tannins added, and into dark-green with tannins and iron mordant), ochre-yellow and vibrant auburn colors with *D. fragrans* (turn ochre-red color in alkaline pH), yellow/beige color with *T. bentzoe and T. cattapa* (turn dark-blue or black with iron mordant), sandy color with *C. equisetifolia* (turn deep-blue with iron mordant, and into terracotta in alkaline pH), yellow and pinkish color with *Coccoloba uvifera*, etc.

## Conclusion

Reunion Island has a multitude of plant species that have potential as a source of new compounds or new raw materials for the dye industries. The current study demonstrated that even if the local sector of natural dyes and uses of plants as source of colorants and pigments are recent to the Island, the biodiversity of Reunion Island has the potential to offer a large choice of alternative sources of colorants and pigments for industry.

After literature review and surveys made on local population, an original classification method has been established to rank the different dye plants collected using different criteria like endemicity, parts used and colors. As a preliminary study, this first survey and classification will direct researchers and industrials to a smaller sample of plants to investigate as the next sources of natural colorants and pigments. It embraces biodiversity and the importance of recovering species that are endemic and/or native to Reunion Island biodiversity which could be used as natural colors. This study also illuminates additional research to be carried out where there is a lack of scientific knowledge and great potential for socio-economic impact through natural colorants from plants origin growing in tropical or sub-tropical regions.

The Indian Ocean region still offers huge potential for discovering new sources of plant-based dyestuffs. The current study is only a first step to demonstrating that the recovery of natural dyes from these tropical regions has a bright future for researchers, industry and consumers. Thus, there is no doubt this is the tip of the iceberg, and many other tropical plant species should be investigated to unveil myriad of other useful natural compounds.

## Supplementary Information

**Additional file 1. Table S1.** List of 194 plant species potentially rich in dyes or pigments inventoried in Reunion Island

## Data Availability

The datasets used and/or analyzed during the current study are available from the corresponding author on reasonable request.
