# Supplementary material for: A survey on the potential contribution of Reunion Island dye plant species diversity to the market demand for bioactive plant-based dyes and pigments
Source: J Ethnobiol Ethnomed. 2023 Mar 25;19:8. doi: 10.1186/s13002-023-00580-w (PMC10039506; doi:10.1186/s13002-023-00580-w)
Supplement: Supplementary file 1 — Additional file 1. Table S1. List of 194 plant species potentially rich in dyes or pigments inventoried in Reunion Island [file 13002_2023_580_MOESM1_ESM.docx]

Table S1. List of 194 plant species potentially rich in dyes or pigments inventoried in Reunion Island

| **Botanical family** | **Scientific name** | **Reunion Island vernacular name** | **Endemic status** | **IUCN Status (2010) / Protection (arrêté du 27102017)** | **Parts used** | **Colors & hues** | **Ethnobotanical survey** |
| --- | --- | --- | --- | --- | --- | --- | --- |
| ACANTHACEAE | Pseuderanthemum carruthersii (Seem.) Guillaumin var. atropurpureum (W. Bull) Fosberg | Plante chocolat | Alien cultivated | NA | Leaves | Green | BINET V. |
| AIZOACEAE | Tetragonia tetragonoides (Pall.) Kuntze | Brède gras | Alien invasive, cultivated | NA | ? | ? | HOARAU-JOLY I. |
| AMARANTHACEAE | Chenopodium album L. | Épinard sauvage (Mau) | Alien naturalized | NA | Young leaves | Green | - |
| AMARYLLIDACEAE | Allium cepa L. | Oignon | Alien cultivated | NA | Bark, peel | Yellow | BINET V. |
| AMARYLLIDACEAE | Allium sativum L. | Ail | Alien cultivated | NA | Bulbs | Green | - |
| ANACARDIACEAE | Anacardium occidentale L. | Noix de cajou | Alien cultivated | NA | ? | ? | PAYET M.-A. |
| ANACARDIACEAE | Mangifera indica L. | Mangue | Alien cultivated, subspontaneous | NA | Leaves | Yellow | GAUVIN G. BINET V. |
| ANACARDIACEAE | Schinus terebinthifolia Raddi | Faux-poivrier | Alien invasive | NA | Leaves | Black | PAYET M.-A |
| ANACARDIACEAE | Spondias dulcis Parkinson | Evi, sakoa | Alien cultivated | NA | Bark | Cachou dye | - |
| ANNONACEAE | Annona muricata L. | Sapoty, Corossol, Corossolier, Sapotille, Sapotiller | Alien cultivated | NA | Bark | ? | - |
| APHLOIACEAE | Aphloia theiformis (Vahl) Benn. | Change-écorce | Native | LC | Bark, stems and leaves | Yellow | - |
| APIACEAE | Petroselinum crispum (Mill.) Nyman ex A.W. Hill var. neapolitanum Danert | Persil | Alien cultivated | NA | Leaves | ? | PAYET M.-A. |
| APOCYNACEAE | Carissa macrocarpa (Eckl.) A. DC. | Prune du Natal | Alien cultivated | NA | Fruits | Red | LUSPOT W. & J.-M |
| APOCYNACEAE | Nerium oleander L. | Laurier rose | Alien cultivated | NA | Flowers | Brown, yellowish | - |
| APOCYNACEAE | Ochrosia borbonica J.F. Gmel. | Bois jaune | Endemic | VU / protected | Bark | Yellow | - |
| ARALIACEAE | Hedera helix L. | Lierre | Alien invasive | NA | Leaves | Jaune clair | PAYET M.-A |
| ARECACEAE | Cocos nucifera L. | Coco | Alien cultivated | NA | Fruit (fiber) | Beige to brown | BINET V., FULSUNGE S. |
| ARECACEAE | Latania lontaroides (Gaertn.) H.E. Moore | Latanier rouge | Endemic | CR / protected | Leaves | Black | LUSPOT W. & J.-M |
| ARECACEAE | Phoenix reclinata Jacq. | Dattier du Sénégal | Alien cultivated | NA | Bark | Brown red | - |
| ASPHODELACEAE | Aloe macra Haw. | Mazambron marron | Endemic | EN / protected | Leaves/sap | Dark yellow - green | GAUVIN G. LUSPOT W. & J.-M. |
| ASPHODELACEAE | Dianella ensifolia (L.) DC. | vacoa nain, reine des bois | Cryptogenic cultivated | DD | Fruits | Blue | - |
| ASPHODELACEAE | Phormium tenax J.R. Forst. et G. Forst. | Vacoa de laine | Alien cultivated | NA | Whole plant | Yellow | GAUVIN G. |
| ASTERACEAE | Bidens pilosa L. | Piquant | Alien invasive (weed) | NA | Whole plant | Orange | FULSUNGE S. |
| ASTERACEAE | Cosmos sulphureus Cav. | Cosmos | Alien cultivated | NA | Whole plant | Orange | FULSUNGE S. |
| ASTERACEAE | Dahlia x hortensis Guill. | Dahlia | Alien cultivated | NA | Flowers | Jaunâtre | PAYET M.-A. |
| ASTERACEAE | Eclipta prostrata (L.) L. | Herbe à l'encre | Alien naturalized | NA | Leaves | Dark greenish-black | - |
| ASTERACEAE | Erigeron sumatrensis Retz. | Mille-feuille | Alien invasive (weed) | NA | Whole plant | Yelllow, khaki green | FULSUNGE S. |
| ASTERACEAE | Hubertia ambavilla Bory var. ambavilla | Ambaville | Endemic | LC | Flower | Yellow-green | FULSUNGE S. |
| ASTERACEAE | Parthenium hysterophorus L. | Camomille-zoiseau | Alien invasive (weed) | NA | Whole plant | Yellow print | BINET V. |
| ASTERACEAE | Psiadia amygdalina (Lam.) Cordem. |  | Endemic | LC | Whole plant | ? | - |
| ASTERACEAE | Psiadia anchusifolia (Poir.) Cordem. | Bouillon blanc | Endemic | LC | Whole plant | ? | - |
| ASTERACEAE | Psiadia argentea (Lam.) Cordem. |  | Endemic | LC | Whole plant | ? | - |
| ASTERACEAE | Psiadia aspera (Bory) Cordem. |  | Endemic | LC | Whole plant | ? | - |
| ASTERACEAE | Psiadia boivinii (Klatt) Rob. | Bouillon blanc | Endemic | LC | Whole plant | ? | - |
| ASTERACEAE | Psiadia callocephala (Bory) Cordem. |  | Endemic | LC | Whole plant | ? | - |
| ASTERACEAE | Psiadia dentata (Cass.) DC. | Ti mangue | Endemic | LC | Whole plant | ? | - |
| ASTERACEAE | Psiadia insignis Cordem. | Sauge | Endemic | NT | Whole plant | ? | - |
| ASTERACEAE | Psiadia laurifolia (Lam.) Cordem. | Bois de tabac | Endemic | LC | Whole plant | ? | - |
| ASTERACEAE | Psiadia melastomatoides (Lam.) A.J. Scott |  | Endemic | LC | Whole plant | ? | - |
| ASTERACEAE | Psiadia montana (Cordem.) Cordem. | Bois marron | Endemic | NT | Whole plant | ? | - |
| ASTERACEAE | Psiadia retusa (Lam.) DC. | La salière | Endemic | VU | Whole plant | ? | - |
| ASTERACEAE | Smallanthus sonchifolius (Poepp.) H.Rob. | Yacón, poire de terre | Alien cultivated | NA | Tubercules | Blue | LUSPOT W. & J.-M. |
| ASTERACEAE | Tagetes erecta L. | Œillet malbar | Alien cultivated | NA | Flowers | ? | GAUVIN G. |
| ASTERACEAE | Tithonia diversifolia (Hemsl.) A. Gray | Fleur-la-fête-des-mères | Alien invasive (weed) | NA | Flowers | Mustard yellow | BINET V. FULSUNGE S. |
| ASTERACEAE | Zinnia elegans Jacq. | Zinnia | Alien cultivated | NA | Flowers | Jaune orangé | PAYET M.-A. |
| BASELLACEAE | Basella alba L. | Brède d'Angole | Alien cultivated | NA | Fruits | Red | - |
| BETULACEAE | Alnus acuminata Kunth | Aulne | Alien cultivated | NA | Bark | Black | - |
| BETULACEAE | Alnus cordata (Loisel.) Duby | Aulne de Corse | Alien cultivated | NA | Bark | ? | - |
| BETULACEAE | Alnus glutinosa (L.) Gaertn. | Aulne glutineux | Alien cultivated | NA | Bark | ? | - |
| BIGNONIACEAE | Jacaranda mimosifolia D. Don | Jacaranda | Alien invasive | NA | Wood | Yellow | - |
| BIXACEAE | Bixa orellana L. | Roucou | Alien cultivated | NA | Seed | Red | LAVERGNE R. HOARAU Y. |
| CACTACEAE | Selenicereus undatus (syn. Hylocereus undatus (Haw.) Britton et Rose) | Pitahaya | Alien cultivated, invasive | NA | Fruit | Red purple | - |
| CALOPHYLLACEAE | Calophyllum inophyllum L. | Tacamaque | Alien cultivated, subspontaneous | NA | Fruits (oil) | Brown | LUSPOT W. & J.-M. |
| CANNABACEAE | Cannabis sativa L. | Zamal | Alien cultivated | NA | Whole plant | ? | - |
| CANNABACEAE | Trema orientalis (L.) Blume | Bois d'andrèze | Alien naturalized | NA | Bark | Brown | HOARAU Y. |
| CAPRIFOLIACEAE | Sambucus canadensis L. | Sureau | Alien cultivated | NA | ? | ? | PAYET M.-A. |
| CASUARINACEAE | Casuarina cunninghamiana Miq. | Filao de Nouvelle Hollande | Alien cultivated | NA | Leaves | ? | - |
| CASUARINACEAE | Casuarina equisetifolia L. | Filao | Alien cultivated, invasive | NA | Bark, leaves | Pink to brown | BINET V. |
| CASUARINACEAE | Casuarina glauca Sieber ex Spreng. | Filao multipliant | Alien cultivated, invasive | NA | Bark | ? | - |
| COMBRETACEAE | Combretum micranthum G.Don | Kinkéliba | Alien cultivated | NA | Leaves | Red | - |
| COMBRETACEAE | Terminalia bentzoe (L.) L. f. subsp. bentzoe | Benjoin | Endemic | CR / protected | Bark | ? | HOARAU Y. LUSPOT W. & J.-M. |
| COMBRETACEAE | Terminalia catappa L. | Badamier | Alien cultivated | NA | Leaves, bark | Brownish | BINET V., LUSPOT W. & J.-M. |
| COMBRETACEAE | Terminalia mantaly H. Perrier | Mantaly | Alien cultivated | NA | Bark, timber | Beige, red | - |
| CORDIACEAE | Cordia africana Lam. | Teck d'Arabie | Alien naturalized | NA | Leaves? | ? | PAYET M.-A. |
| CUNONIACEAE | Weinmannia mauritiana D. Don | Petit bois de tan | Endemic | LC | Bark | ? | - |
| CUNONIACEAE | Weinmannia tinctoria Sm. | Tan rouge, bois de tan, bois de tan rouge, tan blanc, tan des hauts | Endemic | LC | Bark | Red | HOARAU Y., BINET V., GAUVIN G. |
| CUPRESSACEAE | Cryptomeria japonica (L. f.) D. Don | Cryptoméria | Alien cultivated | NA | Leaves | Copper apricot, flesh color | FULSUNGE S. |
| CUPRESSACEAE | Juniperus communis L. | Genièvre | Alien cultivated | NA | Fruits | ? | PAYET M.-A. |
| CYATHEACEAE | Sphaeropteris cooperi (Hook. ex F. Muell.) R.M. Tryon | Fanjan | Alien invasive | NA | Leaves? | ? | PAYET M.-A. |
| DENNSTAEDTIACEAE | Pteridium aquilinum (L.) Kuhn | Fougère aigle | Native | LC | Leaves, fruits | Black | - |
| DIOSCOREACEAE | Dioscorea alata L. | Cambarre de "sang" | Alien cultivated | NA | Tubercules | Mauve | LUSPOT W. & J.-M. |
| ERICACEAE | Agarista salicifolia (Comm. ex Lam.) G. Don | Bois de rempart | Native | LC | Leaf ? Wood ? | ? | HOARAU-JOLY I. |
| ERYTHROXYLACEAE | Erythroxylum laurifolium Lam. | Bois de rongue | Endemic | LC | ? | Red | HOARAU Y. |
| ESCALLONIACEAE | Forgesia racemosa J.F. Gmel. | Bois de Laurent-Martin | Endemic | LC | ? | Rouge | LUSPOT W. & J.-M. |
| EUPHORBIACEAE | Acalypha indica L. | Zoreil chatte, herbe chatte | Alien naturalized | NA | Leaves | Deep green | - |
| EUPHORBIACEAE | Acalypha wilkesiana Müll.Arg. | Foulard | Alien cultivated | NA | Leaves | ? | - |
| EUPHORBIACEAE | Aleurites moluccanus (L.) Willd. | Bancoul | Alien invasive | NA | Bark | Black | - |
| EUPHORBIACEAE | Euphorbia cotinifolia L. | Laitier rouge | Alien cultivated | NA | Leaves | Grey | BINET V. |
| EUPHORBIACEAE | Euphorbia heterophylla L. | Herbe de lait | Alien invasive (weed) | NA | Flowers | Red | - |
| EUPHORBIACEAE | Jatropha curcas L. | Pignon d'Inde | Alien naturalized (cultivated?) | NA | Leaves, stem | Brown | - |
| EUPHORBIACEAE | Ricinus communis L. | Ricin | Alien invasive (weed) | NA | Leaves | Yellow | BINET V. |
| FABACEAE | Acacia dealbata Link | Acacia Bernier, mimosa | Alien invasive | NA | Bark (India), pods (Africa) | Black | - |
| FABACEAE | Acacia mearnsii De Wild. | Acacia | Alien invasive | NA | Bark (India), pods (Africa), flowers, | Yellow, pink beige, grey-brown-black | GAUVIN G., FULSUNGE S. |
| FABACEAE | Acacia podalyriifolia A. Cunn. ex G. Don | Acacia blanc | Alien cultivated | NA | Seeds | ? | PAYET M.-A. |
| FABACEAE | Albizia lebbeck (L.) Benth. | Bwa noir | Alien invasive (cultivated?) | NA | Bark, leaves | Brown, yellowish brown | - |
| FABACEAE | Bauhinia galpinii N.E.Br. | Bauhinia rouge | Alien cultivated | NA | Leaves | Grey-black | FULSUNGE S. |
| FABACEAE | Bauhinia variegata L. | Arbre à orchidées | Alien cultivated | NA | Leaves, bark | Pink to grey dark mole | FULSUNGE S. |
| FABACEAE | Biancaea decapetala (Roth) O. Deg. | Sappan | Alien invasive | NA | Wood, bark | Pink to terracotta, "beige" | BINET V., FULSUNGE S. |
| FABACEAE | Cassia fistula L. | Cytise indien | Alien cultivated | NA | Flowers | Yellow | BINET V. |
| FABACEAE | Clitoria ternatea L. var. ternatea f. ternatea | Liane madame, pistache marron bleu | Alien cultivated, naturalized | NA | Flower | Blue | BINET V. LUSPOT W. & J.-M. |
| FABACEAE | Crotalaria incana L. subsp. purpurascens (Lam.) Milne-Redh. | Crotalaire | Alien subspontaneous | NA | Leaves | Blue, green, black | - |
| FABACEAE | *Crotalaria retusa* L. | Pois rond marron, cascavelle jaune, crotalaire, rattleweed (angl) | Cryptogenic | NA | Whole plant ? | ? | - |
| FABACEAE | Delonix regia (Bojer) Raf. | Flamboyant | Alien cultivated | NA | Flowers | Red | - |
| FABACEAE | Enterolobium cyclocarpum (Jacq.) Griseb. | Zoreil cafre, bois tanniste rouge | Alien cultivated | NA | The bark contains tannins and is used in the tannery to treat fur | Red | - |
| FABACEAE | Faidherbia albida (Delile) A. Chev. | Acacia blanc | Alien cultivated | NA |  | ? | - |
| FABACEAE | Haematoxylum campechianum L. | Bois de campêche | Alien cultivated | NA | Bark, wood | Dark red | - |
| FABACEAE | Indigofera ammoxylum (DC.) Polhill | Bois de sable | Endemic | CR / protected | Wood, leaves??? | Red | LUSPOT W. & J.-M. |
| FABACEAE | Indigofera suffruticosa Mill. | Indigotier sauvage | Alien invasive (weed) | NA | Whole plant | ? | - |
| FABACEAE | Indigofera tinctoria L. | Indigo | Alien naturalized? | NA | Leaves | Blue, green, purple (bleu d'Outre-mer) | LAVERGNE R., LUSPOT W. & J.-M. |
| FABACEAE | Leucaena leucocephala (Lam.) de Wit | Cassi | Alien invasive | NA | Leaves | ? | BINET V. |
| FABACEAE | Libidibia coriaria (Jacq.) Schltdl. | Dividivi | Alien cultivated (?) | NA | Ripe pods | Violet-black | - |
| FABACEAE | Peltophorum pterocarpum (DC.) Backer ex K. Heyne | Flamboyant jaune | Alien cultivated | NA | Bark | Orange, apricot, dark brown | FULSUNGE S. |
| FABACEAE | Pithecellobium dulce (Roxb.) Benth. | Tamarin de l'Inde | Alien naturalized (cultivated?) | NA | Bark | Pink to brown | BINET V. |
| FABACEAE | Senna alata (L.) Roxb. | Quatre épingle | Alien cultivated | NA | Ripe pods (callus) | Brownish, yellowish | - |
| FABACEAE | Senna siamea (Lam.) H.S. Irwin et Barneby | Cassia du Siam | Alien cultivated | NA | Fruit, wood | ? | - |
| FABACEAE | Sophora denudata Bory | Petit tamarin des Hauts | Endemic | EN / protected |  | ? | - |
| FABACEAE | Tephrosia tinctoria (L.) Pers. | Indigo | Alien naturalized | NA | Leaves (Tephrosia candida) | Dark blue (Tephrosia candida) | HOARAU Y., BINET V. |
| FABACEAE | Trigonella foenum-graecum L. | Fenugrec | Alien cultivated | NA | Flowers | Jaune lumineux | PAYET M.-A. |
| FABACEAE | Ulex europaeus L. | Ajonc d'Europe, zépinard des Hauts, Genêt | Alien invasive | NA | Flowers | Pale yellow | FULSUNGE S. |
| FABACEAE | Vachellia nilotica (L.) P.J.H. Hurter et Mabb. | Acacia à gomme | Alien cultivated | NA | Bark | Black, kaki | - |
| FAGACEAE | Castanea sativa Mill. | Châtaigne | Alien cultivated | NA | Fruits ? | ? | - |
| GERANIACEAE | Geranium robertianum L. | Herbe tangue | Alien invasive (weed) | NA | Whole plant | Brown | - |
| HYPERICACEAE | Hypericum lanceolatum Lam. | Fleur jaune | Endemic | LC | Flowers | Bright yellow | GAUVIN G. |
| JUGLANDACEAE | Juglans regia L. | Noyer | Alien cultivated | NA | Bark | Brown green | - |
| LAMIACEAE | Rosmarinus officinalis L. | Romarin | Alien cultivated | NA | Leaves | ? | PAYET M.-A. |
| LAURACEAE | Cassytha filiformis L. | Liane foutafout | Native | LC | Liana | Orangish, yellowish | - |
| LAURACEAE | Cinnamomum burmannii (Nees et T. Nees) Blume | Ti cannelle | Alien cultivated, invasive | NA | Leaves | Red | HOARAU Y. |
| LAURACEAE | Cinnamomum camphora (L.) J. Presl | Camphrier | Alien cultivated, naturalized | NA | Leaves, fruit | Yellowish, reddish | - |
| LAURACEAE | Persea americana Mill. | Avocat | Alien cultivated | NA | Seed, peel extract | Light pink to brown | BINET V. |
| LECYTHIDACEAE | Barringtonia asiatica (L.) Kurz | Bonnet de prêtre | Alien subspontaneous (casual-established), cultivated | NA | Bark | Brown purple | - |
| LECYTHIDACEAE | Foetidia mauritiana Lam. | Bois puant, Bois pian, Bois pion | Endemic | CR / protected | Wood | ? | - |
| LYTHRACEAE | Lawsonia inermis L. | Henné | Alien cultivated | NA | Leaves, seeds | Red to pink, brown | - |
| LYTHRACEAE | Pemphis acidula J.R. Forst. et G. Forst. | Bois matelot | Native | VU | Bark | Red | PAYET M. |
| LYTHRACEAE | Punica granatum L. | Grenade | Alien cultivated, subspontaneous | NA | Bark, peel extract | Black (bark), green (fruit+mordant) or mustard (fruit without mordant) | BINET V., LAVERGNE R. |
| MALVACEAE | Gossypium sp.1 | Cotonnier | Alien naturalized | NA | Leaves & stem | Pink | BINET V. |
| MALVACEAE | Hibiscus sabdariffa L. | Bissap | Alien cultivated | NA | Fruits | Rouge-violacé | LUSPOT W. & J.-M. |
| MALVACEAE | Ruizia cordata Cav | Bois de senteur blanc | Endemic | CR / protected | barks | ? | PAYET M.-A. |
| MALVACEAE | Sparrmannia ricinocarpa (Eckl. et Zeyh.) Kuntze | - | Alien subspontaneous | NA | Leaves | Green | - |
| MALVACEAE | Talipariti tiliaceum (L.) Fryxell | Mova, var | Native? cultivated (N?Q(I?)) | EN / protected | Bark ? | ? | HOARAU Y. |
| MALVACEAE | Theobroma cacao L. | Cacaoyer | Alien cultivated | NA | Fruit or seed | Malachite green | - |
| MALVACEAE | Thespesia populnea (L.) Sol. ex Corrêa | Porché | Alien? (Cryptogenic?) cultivated (Q(K?N?)) | DD | Fruits, flowers, bark | Red to light brown | HOARAU Y. |
| MELIACEAE | Khaya senegalensis (Desr.) A. Juss. | Acajou du Sénégal | Alien cultivated | NA | fruit (teguments) | Pink | BINET V. |
| MELIACEAE | Toona ciliata M. Roem. | Cedrela | Alien cultivated | NA | Bark, flower | Reddish, yellowish | - |
| MORACEAE | Artocarpus heterophyllus Lam. | Jacque | Alien cultivated, subspontaneous | NA | Wood ? | Yellow | GAUVIN G., HOARAU-JOLY I., BINET V. |
| MORACEAE | Ficus densifolia Miq. | Affouche | Endemic | LC | Wood | Rouge | LUSPOT W. & J.-M. |
| MORACEAE | Ficus rubra Vahl | Affouche rouge | Native | LC | Wood | Rouge | LUSPOT W. & J.-M. |
| MORACEAE | Ficus sycomorus L. | Sycomore | Alien cultivated | NA | Bark | Black | - |
| MORACEAE | Morus alba L. var. indica (L.) Bureau | Mûrier | Alien invasive, cultivated | NA | Bark | ? | HOARAU-JOLY I., LUSPOT W. & J.-M. |
| MORINGACEAE | Moringa oleifera Lam. | Mouroungue | Alien cultivated | NA | Wood | Blue | - |
| MUSACEAE | Musa acuminata Colla | Banane-figue | Alien cultivated | NA | Fruit sap | Black | GAUVIN G., HOARAU-JOLY I. |
| MYRTACEAE | Eucalyptus robusta Sm. |  | Alien cultivated | NA | Leaves | Grey-Black | FULSUNGE S. |
| MYRTACEAE | Eucalyptus sp. | Eucalyptus | Alien cultivated | NA | Leaves, bark | ? | BINET V. |
| MYRTACEAE | Eucalyptus sp. (Etang-Salé) |  | Alien cultivated | NA | Leaves | Gris vert kaki au noir gris | FULSUNGE S. |
| MYRTACEAE | Myrtus communis L. | Myrte | Alien cultivated | NA | Leaves | ? | PAYET M.-A. |
| MYRTACEAE | Psidium cattleyanum Sabine | Goyavier | Alien invasive | NA | Wood, fruit | Reddish (fruit), black (wood) | LAVERGNE C., BINET V., FULSUNGE S. |
| MYRTACEAE | Psidium guajava L. | Goyave | Alien cultivated, naturalized | NA | Barks | ? | PAYET M.-A. |
| MYRTACEAE | Syzygium cumini (L.) Skeels | Jamblon | Alien invasive, cultivated | NA | Fruit | Purple, yellowish | HOARAU-JOLY I. W. & J.-M. |
| OLACACEAE | Olax psittacorum (Lam.) Vahl | Corce rouge, bois d'effort | Endemic | VU | Bark | ? | LAVERGNE C. |
| OLEACEAE | Olea lancea Lam. | Bois d'olive blanc | Native | LC | Leaves | ? | PAYET M.-A. |
| OROBANCHACEAE | Alectra sessiliflora (Vahl) Kuntze |  | Native? | LC | Flowers, roots | Yellow | - |
| PANDANACEAE | Pandanus utilis Bory | Vacoa | Native (endemic?), cultivated Q(I?) | LC | Terminal bud, leaves | Yellow | GAUVIN G., LUSPOT W. & J.-M. |
| PAPAVERACEAE | Argemone mexicana L. | Chardon | Alien invasive (weed) | NA | Sap | Yellowish sap | - |
| PASSIFLORACEAE | Passiflora edulis Sims | Grenadine | Alien cultivated, naturalized | NA | Fruits (teguments) | ? | PAYET M.-A. |
| PASSIFLORACEAE | Passiflora suberosa L. (ou P. pallida L.) | Grain d'encre | Alien invasive (weed) | NA | Fruit | Dark purple | GAUVIN G. |
| PHYLLANTHACEAE | Antidesma madagascariense Lam. | Bois de cabri blanc | Native | LC | Fruits | bluish-purple | HOARAU H. |
| PHYLLANTHACEAE | Securinega durissima J.F. Gmel. | Bois dur | Native | LC | Bark | Reddish sap | LAVERGNE C. |
| PHYTOLACCACEAE | Phytolacca americana L. | Faux-vin | Alien invasive (weed) | NA | Fruit | Ivory, grey | LAVERGNE R., BINET V., GAUVIN G., HOARAU-JOLY I. |
| PLANTAGINACEAE | Plantago major L. | Plantin | Alien naturalized | NA | Leaves | ? | PAYET M.-A. |
| POLYGONACEAE | Antigonon leptopus Hook. et Arn. | Liane antigone | Alien invasive | NA | Leaves (stems and flowers) | Brown, green | BINET V., HOARAU Y., FULSUNGE S. |
| POLYGONACEAE | Coccoloba uvifera (L.) L. | Raisin de mer | Alien cultivated | NA | Fruit, leaves | Purpleish | BINET V., HOARAU Y., LUSPOT W. & J.-M. |
| POLYGONACEAE | Persicaria chinensis (L.) H. Gross | Liane rouge | Alien invasive (weed) | NA | Leaves | Grey black | GAUVIN G. |
| POLYGONACEAE | Rumex sp. | Oseille | Alien invasive (weed) | NA | Whole plant | Pink beige, grey-beige | FULSUNGE S. |
| POLYPODIACEAE | Phymatosorus scolopendria (Brum f.) Pic Serm | Patte lézard | Native | LC | Leaves | ? | PAYET M.-A. |
| PROTEACEAE | Grevillea robusta A. Cunn. ex R. Br. | Grévillaire | Alien invasive (cultivated?) | NA | Bark, leaves | Pink (bark), yellow (leaves), green (leaves) | BINET V., FULSUNGE S. |
| PROTEACEAE | Macadamia integrifolia Maiden et betche | Noix de Macadamia | Alien cultivated | NA | ? | ? | PAYET M.-A. |
| RHAMNACEAE | Ziziphus mauritiana Lam. | Jujube | Alien cultivated | NA | Bark, leaves | Light brown | - |
| ROSACEAE | Eriobotrya japonica (thunb.) Lindl. | Bibasse | Alien cultivated, invasive | NA | Barks | Pinkish | PAYET M.-A. |
| RUBIACEAE | Antirhea borbonica J.F. Gmel. | Bois d'osto | Native | LC | Leaves, bark | Green | - |
| RUBIACEAE | Bertiera borbonica A. Rich. ex DC. | Bois de raisin | Endemic | DD | Fruits | Bluish | LAVERGNE C. |
| RUBIACEAE | Bertiera rufa DC. | Bois de raisin | Endemic | LC | Fruits | Bluish | LAVERGNE C. |
| RUBIACEAE | Cremaspora triflora (Thonn.) K. Schum. | - | Alien cultivated | NA | Fruits, seeds | Black blue, red | - |
| RUBIACEAE | Danais fragrans (Lam.) Pers. | Liane jaune | Native | LC | Roots | Orangish red | LAVERGNE R. |
| RUBIACEAE | Galium aparine L. | Garance, gaillet gratteron | Alien invasive | NA | Roots | pink-red | BINET V. |
| RUBIACEAE | Ixora coccinea L. | Ixora | Alien cultivated | NA | Flowers | Pink, grey | - |
| RUBIACEAE | Morinda citrifolia L. | Malaye, bois tortue | Native cultivated | DD | Roots | Yellow | BINET V. |
| RUBIACEAE | Mussaenda arcuata Poir. | Lingue café | Native | LC | Roots, flowers | Red | - |
| RUBIACEAE | Psychotria borbonica (J.F. Gmel.) Razafim. et B. Bremer | Bois cassant | Endemic | LC | Stem and leaves | ? | - |
| RUBIACEAE | Uncaria gambir (W. Hunter) Roxb. | Cachou-gambir (gambier cachou) | Alien cultivated (?) | NA | Leaves | Cachou dye | - |
| RUTACEAE | Aegle marmelos (L.) Corrêa | Vilvone, baël, orange miel | Alien cultivated | NA | Fruit rinds | Camel | FONTAINE C. |
| SAPINDACEAE | Dodonaea viscosa Jacq. | Bois d'arnette | Native | LC | Leaves, bark | Yellow-green (leaves), brown (bark) | FULSUNGE S., LUSPOT J.-M. & W. |
| SAPINDACEAE | Doratoxylon apetalum (poir.) Radlk | Bois de gaulette | Native | LC | Leaves | ? | PAYET M.-A. |
| SAPINDACEAE | Litchi chinensis Sonn. | Letchi | Alien cultivated | NA | Barks, fruit skin | Brownish | PAYET M.-A., LUSPOT W. & J.-M. |
| SAPINDACEAE | Molinaea alternifolia Willd. | Tan Georges | Endemic | LC | ? | ? | HOARAU Y. |
| SAPOTACEAE | Mimusops balata (Aubl.) C.F. Gaertn. | Grand natte, natte à grandes feuilles, bois de nate, bois de natte | Endemic | LC | Bark | Brownish | LAVERGNE R. |
| SOLANACEAE | Capsicum frutescens L. | Piment | Alien cultivated | NA | Fruit | Red | - |
| SOLANACEAE | Cestrum nocturnum L. | Jasmin de nuit | Alien cultivated | NA | Flowers (pistil) | ? | BINET V. |
| SOLANACEAE | Physalis peruviana L. | Poc-poc, Groseille du Cap, Poc poc | Alien naturalized | NA | Whole plant | ? | - |
| SOLANACEAE | Solanum americanum Mill. | Brède morel | Alien invasive (weed), cultivated | NA | Fruit | Blackish | LAVERGNE C. |
| SOLANACEAE | Solanum anguivi Lam. | Petite anghive | Alien cultivated | NA | Leaves | Green | - |
| STELITZIACEAE | Ravenala madagascariensis Sonn. | Ravenale, arbre du voyageur | Alien cultivated | NA | Fruit | Turquoise | ANDRIAMANANTENA M., |
| THEACEAE | Camellia sinensis (L.) Kuntze | Thiéier | Alien cultivated | NA | Leaves | Beige to dark beige | PAYET M.-A. |
| VERBENACEAE | Stachytarpheta urticifolia Sims | Queue de rat | Alien naturalized | NA | ? | ? | PAYET M.-A. |
| VITACEAE | Leea guineensis G. Don | Bois de sureau | Native | LC | Barks | Red | - |
| ZINGIBERACEAE | Curcuma longa L. | Safran-pays | Alien cultivated | NA | Rhizome | Dark yellow | LAVERGNE R. |
